# The Role of Sarcolipin in Muscle Non-shivering Thermogenesis

**DOI:** 10.3389/fphys.2018.01217

**Published:** 2018-09-27

**Authors:** Naresh C. Bal, Sanjaya K. Sahoo, Santosh K. Maurya, Muthu Periasamy

**Affiliations:** ^1^KIIT School of Biotechnology, KIIT University, Bhubaneswar, India; ^2^Sanford Burnham Prebys Medical Discovery Institute at Lake Nona, Orlando, FL, United States

**Keywords:** sarcolipin (SLN), nonshivering thermogenesis (NST), skeletal muscle, SERCA (sarco(endo)plasmic reticulum calcium ATPase), calcium transport across biological mem branes

## Introduction

The opinion review by Campbell and Dicke questioned if sarcolipin (SLN) is involved in adaptive thermogenesis especially cold adaptation, while agreeing to SLN-mediated heat production (Campbell and Dicke, 2018). Several interesting questions were raised concerning the data implicating SLN in muscle nonshivering thermogenesis (NST). Here, we intend to clarify to the research community that SLN-mediated heat production and adaptive thermogenesis are inseparable. This is because heat production by SLN-SERCA interaction is a biochemical mechanism, whose physiological implication is adaptive thermogenesis. Recent studies from several groups have identified SLN as an important regulator of SERCA pump (Smith et al., 2002; Tupling et al., 2002; Asahi et al., 2003; MacLennan et al., 2003; Vangheluwe et al., 2005; Mall et al., 2006; Morita et al., 2008; Gorski et al., 2013, 2017; Toyoshima et al., 2013; Winther et al., 2013; Gamu et al., 2014, 2015; Montigny et al., 2014; Fajardo et al., 2015). X-ray Crystallographic studies notably from two groups Toyoshima and Nissen have provided the molecular details of SLN binding to SERCA, inside the TM groove (Toyoshima et al., 2013; Winther et al., 2013). Studies using genetically altered mouse models provided convincing data that SLN is important for muscle thermogenesis and metabolism (Bombardier et al., 2013; Sahoo et al., 2013; Maurya et al., 2015; Rowland et al., 2015a; Bal et al., 2017). Although the molecular details of SLN function in muscle physiology continues to evolve, there is critical evidence that SLN is a key regulator muscle NST.

## SLN is an uncoupler of SERCA pump leading to increased heat production

SERCA is a Ca^2+^ ion transport pump in muscle and its activity is regulated by small peptides including phospholamban (PLB-52 aa) and sarcolipin (SLN-31 aa), whose interaction with SERCA alters the dynamics of Ca^2+^ cycling (Odermatt et al., 1998; Maclennan, 2004; Bhupathy et al., 2007; Traaseth et al., 2008; Periasamy et al., 2017). SLN expression is muscle specific, and its expression level is highly regulated during muscle development and disease states (Vangheluwe et al., 2005; Babu et al., 2007; Pant et al., 2015). PLB is known as an affinity modulator of SERCA pump for Ca^2+^. Interestingly PLB binds to SERCA only to the Ca^2+^-free E2-state, whereas SLN can bind to Ca^2+^-bound SERCA and can remain bound during the Ca^2+^-transport cycle. The presence of SLN decreases the Vmax of Ca^2+^-uptake but not the amount of ATP hydrolyzed (Sahoo et al., 2013, 2015; Shaikh et al., 2016). Anthony Lee and his colleagues were the first to suggest that SLN binding to SERCA could promote uncoupling of SERCA-mediated ATP hydrolysis from Ca^2+^ transport (Smith et al., 2002; Mall et al., 2006). They further showed that Ca^2+^ accumulation in the vesicles decreased, but the heat released by SERCA increased in the presence of SLN. Based on this, they suggested that SLN binding to SERCA prevents release of Ca^2+^ into the lumen and promotes slippage of Ca^2+^ back to the cytosol. The efficiency of SLN uncoupling of SERCA may depend on several factors including a) the ratio of SLN to SERCA; b) cytosolic Ca^2+^-concentration; c) SR luminal Ca^2+^-load; d) ATP availability in the local milieu and e) other factors yet to be defined. A recent study by Nowack et al provides strong evidence that SLN-to-SERCA ratio is utilized by pigs to recruit skeletal muscle thermogenesis independent of shivering. Using muscle SR vesicles expressing high SLN, we determined that the efficiency of SLN uncoupling is not 100% but amounts to no more than 30% of SERCA which suggests while SLN uncoupling function does not interfere with normal muscle function since SERCA pump is in excess. Considering the fact that muscle forms more than 40% of the body weight of all endothermic vertebrates, even a small fraction of heat coming from SLN-mediated uncoupling of SERCA function can have a significant impact on thermoregulation.

## The structural features involved in SLN binding and uncoupling of SERCA

The detailed mechanism of SLN uncoupling is an evolving area of research, but many structural features of SLN/SERCA interaction were identified by protein cross-linking and using SERCA/SLN co-crystals. The X-ray crystals showed that SLN binds to the SERCA TM groove formed by TMs 2, 6, and 9 (Sahoo et al., 2015; Shaikh et al., 2016) however, these studies could not localize the N-terminus of SLN. Previous studies have shown that the C-terminal residues of SLN (Tyr29 and Tyr31), are important for regulation of SERCA (Odermatt et al., 1998) and or SLN localization and interaction with SERCA (Butler et al., 2011). Employing protein chimeras of SLN and PLB, we determined the function of individual domains in uncoupling. We found addition of SLN C-terminus to PLB can increase PLB binding affinity to SERCA but does not promote uncoupling of SERCA (Sahoo et al., 2015). Recent studies from our laboratory suggest that the N-terminus of SLN is important for SLN function (Sahoo et al., 2015). The short N-terminus varies across species and ^2^ERSTQE sequence in rodents change to ^2^ERSTRE in rabbit and to ^2^GINTRE in primates. It contains a conserved Thr5 residue that has been proposed to be a site of Phosphorylation (Bhupathy et al., 2009). Using mutagenesis and chimeric proteins made between SLN and PLB, we have shown that SLN requires its N-terminus for its uncoupling function. Deletion of the N-terminal residues (MERSTQ) caused SLN to constitutively bind to the SERCA groove but did not possess the uncoupling ability. Recently, Autry et al speculated that the negatively charged Glutamate residues are critical for the uncoupling function (Autry et al., 2016). This suggests that mere occupation of the groove is insufficient for uncoupling function but requires the dynamic interaction between the N-terminus, the TM, and the C-terminus of SLN with SERCA for its regulation.

## SLN plays a role in both shivering and non-shivering thermogenesis

The opinion review questioned if SLN is recruited during adaptive thermogenesis. While skeletal muscle has been known to generate heat through shivering, the role of NST in muscle has not been fully appreciated until recently. Studies in birds and mammals suggest that cold adaptation involves upregulation of SR proteins in addition to metabolic remodeling of the muscle (Rowland et al., 2015a; Bal et al., 2016, 2017). Both shivering and NST rely on SR Ca^2+^-cycling, so invariably recruits SLN and SERCA, therefore SLN-dependent heat production is a component of both shivering and NST mechanisms. It is interesting to point out that during shivering the level of Ca^2+^-leak from the SR is very high which would recruit myosin-ATPase as well. Further, as discussed earlier high cytosolic Ca^2+^-concentration reduce SLN interaction/uncoupling of SERCA, so SLN function during shivering might be an important but minor. During prolonged cold adaptation animals switch from shivering to NST mechanisms; which is activated through an increase in cytosolic Ca^2+^ either via RYR1-mediated Ca^2+^-leak or Ca^2+^-entry through channels in the plasma membrane. It is known that cold adapted mice including UCP1 knockout (UCP1-KO) do not shiver when challenged with cold which argues that NST in muscle is primarily responsible for heat production. This idea is further supported by two independent observations made recently. First, mice with surgical ablation of interscapular BAT acclimated to cold significantly upregulate SLN expression in their skeletal muscles (Bal et al., 2016). Similarly, SLN is upregulated in skeletal muscles of UCP1-KO mice, even when exposed to mild cold (Bal et al., 2017). Despite these progress, there are several gaps in our knowledge including how SLN-based NST is activated, whether this involves neurohormonal signaling to trigger Ca^2+^-entry through plasma membrane channels and/or release from the SR. At this time the major mechanism appears to be RYR1-mediated Ca^2+^-leak that can trigger muscle heat production during cold adaptation (Dumonteil et al., 1993, 1995; Aydin et al., 2008; Bal et al., 2016).

## SLN is recruited in diet induced adaptive thermogenesis

Compared to rodents, in large adult mammals including rabbits, dogs and humans, BAT-mediated thermogenesis is negligent and SLN levels are much more abundant (Babu et al., 2007; Rowland et al., 2015b). To mimic SLN expression found in large mammals, we overexpressed SLN using skeletal α-actin gene promoter (Maurya et al., 2015). Sln over-expression (Sln-OE) did not affect muscle function but increased BMR. When pair-fed, Sln-OE mice showed a higher rate of oxygen consumption and lost its fat mass, whereas Sln-KO mice consumed less oxygen and gained fat mass. We further showed that Sln-OE mice were resistant to high fat diet induced obesity and were protected from lipotoxicity in muscle, suggesting higher SLN leads to enhanced energy expenditure through increased mitochondrial biogenesis (Maurya and Periasamy, 2015; Maurya et al., 2015; Sopariwala et al., 2015). A recent study by Maurya et al, has demonstrated that SLN-mediated Ca^2+^-signaling promote mitochondrial health and muscle metabolism (Maurya et al., 2018). On the other hand, a manuscript published by Butler et al. shows that SLN could not be detected in rodent muscle and they were unable to increase SLN expression through transgenesis in muscle (Butler et al., 2015). We believe that there might be technical issues with SLN detection since this protein is highly hydrophobic and of very small size (~3.5 kDa) and the use different mouse strain could also contribute to this discrepancy since our Sln-OE mouse model is in C57Bl6/j background. It has been shown that cold intolerance of UCP1-KO varies significantly between different mouse strains (Hofmann et al., 2001).

## SLN based muscle NST can compensate for the loss of BAT function in mice

Recent studies also highlighted that in rodents muscle based thermogenesis is recruited in addition to BAT during cold adaptation (Monemdjou et al., 2000; Anunciado-Koza et al., 2008, 2011; Bruton et al., 2010; Shabalina et al., 2010; Rowland et al., 2015a; Bal et al., 2016, 2017; Stanford et al., 2018). Barbara Cannon's group showed that cold adaptation of UCP1-KO mice affect skeletal muscle metabolism (Aydin et al., 2008), but argued that this is primarily due to muscle shivering. Interestingly, several groups have found that Ca^2+^-handling in the skeletal muscle is altered in cold adapted UCP1-KO mice. We further investigated the importance of muscle NST in UCP1-KO mice by exposing them to different regimens of cold challenge. We found that skeletal muscles from gradually cold adapted UCP1-KO mice upregulated SLN expression, become redder with increased mitochondrial content, succinate dehydrogenase (SDH) activity as opposed to SLN–KO mice. Interestingly, SLN knockout mice adapted to cold showed significant upregulation of UCP1 and higher mitochondrial content in BAT suggesting that SLN-KO mice increasingly rely on BAT-based NST to compensate for decreased muscle thermogenesis.

Using double knockout (DKO) mice for SLN and UCP1, we studied their ability to adapt to cold. These DKO mice were unable to withstand acute cold (4°C) exposure, while UCP1-KO exhibit only 30% cold sensitivity (Rowland et al., 2015a). Further, we do not believe shivering is the sole mechanism for the survival of UCP1-KO mice during cold adaptation. To gain insight into this question we have performed cold adaptation studies on UCP1-KO mice employing gradual decrease (2.0°C/day) and acute decrease (5°C/day) of ambient temperature of mice reared at 29°C. During this cold exposure, the UCP1-KO show visible shivering only for a short period. When acutely shifted from 29 to 16°C and from 16 to 4°C, mice showed visible shivering for up to 2 days but recognizable shivering decreases and stops by 4th day. Based on these studies we suggest that, subsequent to an initial phase of shivering, SLN-based NST becomes the primary mechanism of thermogenesis as continuous shivering will lead to muscle damage.

## SLN might be the major determinant of muscle thermogenesis in birds and BAT-Deficient mammals

**Figure 1:**
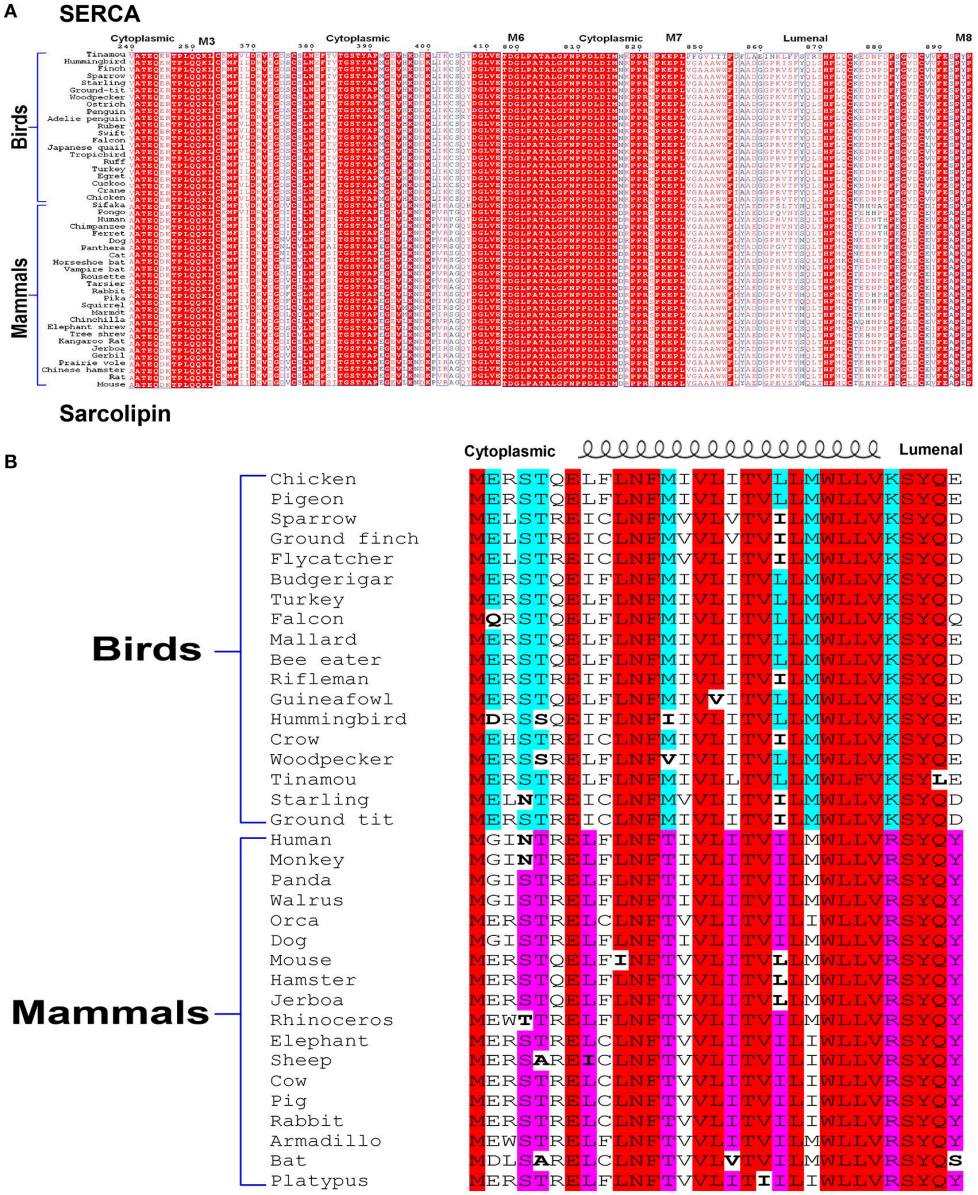
Alignment of SERCA **(A)** and SLN **(B)** sequences. Comparison of SERCA sequences from various species of birds and mammals shows that the cytosolic residues that may potentially interact with SLN do not have strict conservation. SLN sequences also show divergence more on the N-terminal cytosolic and C-terminal luminal side. Like other transmembrane proteins, transmembrane residues of both the proteins are more strictly conserved. Therefore, avian SLN may be better suited to uncouple avian SERCA.

From the studies described here, it is evident that SLN plays a very important role in thermogenesis, both in shivering and muscle NST. Campbell and Dicke rightly pointed out that all our studies have been in the mouse model. We agree that we have not studied large animal models and/or avian species. However, we want to emphasize that we have compared SLN protein expression in mice vs. large animals (like rabbit, dog), and humans (Babu et al., 2007; Rowland et al., 2015b). SLN is highly abundant in all the muscles of larger mammals including human and SLN has a better intracellular milieu for its physiological function in oxidative fibers [discussed by Nowack et al. (2017)]. Based on the expression profile, we do not believe that SLN can have a completely different physiological role in larger mammals compared to mice. We were unable to study SLN expression in birds using our antibody as it detects the C-terminal sequence “RSYQY” that is not conserved in birds (KSYQX). However SLN is highly conserved in the TM region, suggesting avian SLN bind to the same groove of SERCA. The C-terminus of avian SLN does not have conserved sequence but the N-terminus has conserved Glutamate at 2nd and 7th position in sparrow, chicken and pigeon important for uncoupling function (Sahoo et al., 2015; Autry et al., 2016). It is to be noted that avian SERCA bears several residue substitutions especially in the cytosolic and luminal loop regions that SLN can potentially interact as shown in Figure 1. Hence, difference in SLN sequence as seen in Figure 1, might provide better anchoring leading to increased function and not loss of function as indicated by the authors. Complete sequence alignment of SERCA is presented as supplemetal data Data Sheet 1. It shows that transmembrane helices are highly conserved, which provides the groove for SLN to bind to SERCA.

## Perspectives and conclusions

While shivering is an important mechanism of heat production in birds and mammals, prolonged shivering would compromise many physiological functions of an animal and even compromise survival in the wild. In addition mild temperature fluctuations do not activate shivering but rely primarily on NST mechanisms. It is of interest and surely significant that SLN is expressed throughout the vertebrate, from fish to man and might be involved in more fundamental role of local heat production. The role of SR Ca^2+^-cycling in muscle thermogenesis has been documented in the “heater organ”: a modified extraocular muscle found in deep sea fishes (Block and Franzini-Armstrong, 1988; Morrissette et al., 2003) and in malignant hyperthermia, a disease where excessive Ca^2+^-leaks from RyR coupled with chronic SERCA Ca^2+^-cycling leading to pathological heat production (Gommans et al., 2002; Rossi and Dirksen, 2006). These examples do suggest that SR can be adapted as a heat producing mechanism. While thermogenesis is often thought as important for whole body temperature regulation, heat production in muscle plays an intrinsic role in peak muscle performance and more importantly survival, especially when faced with a predator–prey situation. True endothermy is seen only in birds and mammals, yet it relies on BAT in only one taxa, the eutherian mammals. The other extant mammals, monotremes and marsupials, lack BAT but are competent endotherms. Birds too lack BAT but manage endothermy at the highest body temperatures of any, 40°C and more. These all must achieve their endothermy by relying on regulatory NST sourced in the skeletal muscle. [reviewed by Nowack et al. (2017)]. Uncoupling mechanisms or futile mechanisms are not entirely new but exist at the expense of increased energy demand. Regulation of SERCA by small peptides including PLB, SLN, myoregulin and others have provided new roles for SERCA pump activity in muscle Physiology (Anderson et al., 2015). We suggest that SLN uncoupling of SERCA activity evolved during vertebrate evolution to support heat production in muscle important for both muscle performance and thermogenesis. Although the molecular details how SLN binding promotes uncoupling of SERCA and recruited under different pathophysiological conditions remain to be solved, the recent studies provide strong evidence that SLN is important for muscle thermogenesis.

## Author contributions

All authors drafted the manuscript together, critically revised the work and approved the final version. NB prepared the figures.

### Conflict of interest statement

The authors declare that the research was conducted in the absence of any commercial or financial relationships that could be construed as a potential conflict of interest.
