# Supplementary material for: The Role of Sarcolipin in Muscle Non-shivering Thermogenesis
Source: Front Physiol. 2018 Sep 27;9:1217. doi: 10.3389/fphys.2018.01217 (PMC6170647; doi:10.3389/fphys.2018.01217)
Supplement: Supplementary file 1 [file Data_Sheet_1.PDF]

	1 10 20 30 40 50 60
Tinamou_SERCA	MENAHTKTVEEVLAYFGVNESTGLSLEQVKKLKEKWGSNELPAEEGKTLLELVIEQFEDL
Hummingbird_SERCA	MENAHTKTVEEVLAYFGVNESTGLSLEQVKKLKEKWGSNELPAEEGKTLLELVIEQFEDL
Manakin_SERCA	MENAHTKTVEEVLAYFGVNESTGLSLEQVKKLKEKWGSNELPAEEGKTLLELVIEQFEDL
Finch_SERCA1	MENAHTKTVEEVLAYFGVNESTGLSLEQVKKLKEKWGSNELPAEEGKTLLELVIEQFEDL
Sparrow_SERCA	MENAHTKTVEEVLAYFGVNESTGLSLEQVKKLKEKWGSNELPAEEGKTLLELVIEQFEDL
Starling_SERCA	MENAHTKTVEEVLAYFGVNESTGLSLEQVKKLKEKWGSNELPAEEGKTLLELVIEQFEDL
Tibetan_ground-tit_SERCA	MENAHTKTVEEVLAYFGVNESTGLSLEQVKKLKEKWGSNELPAEEGKTLLELVIEQFEDL
Ground-finch_SERCA	MENAHTKTVEEVLAYFGVNESTGLSLEQVKKLKEKWGSNELPAEEGKTLLELVIEQFEDL
Downy_woodpecker_SERCA	MENAHTKTVEEVLAYFGVNESTGLSLEQVKKLKEKWGSNELPAEEGKTLLELVIEQFEDL
Struthio_camelus_SERCA	MENAHTKTVEEVLAYFGVNESTGLSLEQVKKLKEKWGSNELPAEEGKTLLELVIEQFEDL
Penguin_SERCA	MENAHTKTVEEVLAYFGVNESTGLSLEQVKKLKEKWGSNELPAEEGKTLLELVIEQFEDL
Adelie_penguin_SERCA	MENAHTKTVEEVLAYFGVNESTGLSLEQVKKLKEKWGSNELPAEEGKTLLELVIEQFEDL
Ruber_ruber_SERCA	MENAHTKTVEEVLAYFGVNESTGLSLEQVKKLKEKWGSNELPAEEGKTLLELVIEQFEDL
Swift_SERCA	MENAHTKTVEEVLAYFGVNESTGLSLEQVKKQKEKWGSNELPAEEGKTLLELVIEQFEDL
Falcon_SERCA	MENAHTKTVEEVLAYFGVNESTGLSLEQVKKLKEKWGSNELPAEEGKTLLELVIEQFEDL
Japanese-quail_SERCA	MENAHTKTVEEVLAYFGVNESTGLSLEQVKKLKEKWGSNELPAEEGKTLLELVIEQFEDL
White-tailed_tropicbird_SERCA	MENAHTKTVEEVLAYFGVNESTGLSLEQVKKLKEKWGSNELPAEEGKTLLELVIEQFEDL
Ruff_SERCA	MENAHTKTVEEVLAYFGVNESTGLSLEQVKKLKEKWGSNELPAEEGKTLLELVIEQFEDL
Turkey_SERCA	MENAHTKTVEEVLAYFGVNESTGLSLEQVKKLKEKWGSNELPAEEGKTLLELVIEQFEDL
Little-egret_SERCA	MENAHTKTVEEVLAYFGVNESTGLSLEQVKKLKEKWGSNELPAEEGKTLLELVIEQFEDL
Cuckoo_SERCA	MENAHTKTVEEVLAYFGVNESTGLSLEQVKKLKEKWGSNELPAEEGKTLLELVIEQFEDL
Crowned-crane_SERCA	MENAHTKTVEEVLAYFGVNESTGLSLEQVKKLKEKWGSNELPAEEGKTLLELVIEQFEDL
Chicken_SERCA1	MENAHAKTAEECLAFFGVNESVGLSGEQVRRALEKYGHNELPAEEGKTIWELVVEQFEDL
Sifaka_SERCA1	MDAAHAKTTEECLAYFGVSETTGLTPDQVKRHLEKYGPNELPAEEGKSLWELVVEQFEDL
Pongo_SERCA1	MEAAHAKTTEECLAYFGVSETTGLTPDQVKRNLEKYGPNELPAEEGKTLWELVIEQFEDL
Human_SERCA1	MEAAHAKTTEECLAYFGVSETTGLTPDQVKRNLEKYGLNELPAEEGKTLWELVIEQFEDL
Chimpanzee_SERCA1	MEAAHAKTTEECLAYFGVSETTGLTPDQVKRNLEKYGLNELPAEEGKTLWELVIEQFEDL
Domestic_ferret_SERCA1	MEAAHSKTTEECLAYFGVSETTGLSPDQVKRHLEKYGPNELPAEEGKSLWELVVEQFEDL
Dog_SERCA1	MEAAHSKTTEECLAYFGVSETTGLSPDQVKRHLEKYGPNELPAEEGKTLWELVIEQFEDL
Panthera_SERCA1	MEAAHSKTTEECLAYFGVSETTGLTPDQVKRHLEKYGPNELPAEEGKSLWELVIEQFEDL
Cat_SERCA1	MEAAHSKTTEECLAYFGVSETTGLTPDQVKRHLEKYGPNELPAEEGKSLWELVIEQFEDL
Horseshoe-bat_SERCA1	MEGAHSKSTEECLAYFGVSETTGLTPDQVKRHLEKYGPNELPAEEGKSLWELVVEQFEDL
Vampire_bat_SERCA1	MEAAHSKTTEECLSYFGVSETTGLTPDQVKRHLEKYGPNELPAEEGKSLWELVVEQFEDL
Egyptian_rousette_SERCA1	MEAAHSKTTEECLAYFGVSETTGLTLDQVKRHLEKYGPNELPAEEGKSLWELVIEQFEDL
Philippine_tarsier_SERCA1	MEAAHAKTTEECLAHFGVSETTGLTPDQVKRHLEKYGPNELPAEEGKSLWELVVEQFEDL
Rabbit_SERCA1	MEAAHSKSTEECLAYFGVSETTGLTPDQVKRHLEKYGHNELPAEEGKSLWELVIEQFEDL
American_pika_SERCA1	MEAAHSKSTEECLAYFGVSETTGLTPDQVKRHLEKYGPNELPAEEGKSLWELVIEQFEDL
Thirteen-lined_ground_squirrel_SERCA1	MEAAHAKTTEECLAYFGVSETTGLSQDQVKRHLEKYGPNELPAEEGKSLWELVVEQFEDL
Alpine_marmot_SERCA1	MEAAHAKSTEECLAYFGVSETTGLTPDQVKRHLEKYGPNELPAEEGKSLWELVIEQFEDL
Chinchilla_SERCA1	MEAAHAKTTEECLAYFGVSETTGLTPDQVKRHLEKYGPNELPAEEGKSLWELVVEQFEDL
Cape_elephant-shrew_SERCA1	MEAAHSKTTEECLAHFGVSETTGLTPDQVKRHLEKYGPNELPAEEGKSLWELVVEQFEDL
Chinese_	MEAAHSKSTEECLAYFGVSETTGLTPDQVKRHLEKYGPNELPAEEGKSLWELVVEQFEDL
Kangaroo_rat_SERCA1	MEAAHSKSTEECLAYFGVSETTGLTPDQVKRHLEKYGPNELPAEEGKSLWELVVEQFEDL
Egyptian_jerboa_SERCA1	MEAAHAKTTEECLAYFGVSETTGLTLDQVKRHLEKYGPNELPAEEGKSLWELVVEQFEDL
Mongolian_gerbil_SERCA1	MEAAHSKSTEECLSYFGVSETTGLTPDQVKRHLEKYGPNELPAEEGKSLWELVIEQFEDL
Prairie_vole_SERCA1	MEAAHSKSTEECLSFFGVSETTGLTPDQVKRHLEKYGPNELPAEEGKSLWELVVEQFEDL
Chinese	MEAAHSKSTEECLSYFGVSETTGLTPDQVKRHLEKYGPNELPAEEGKSLWELVVEQFEDL
Rattus_SERCA1	MEAAHSKSTEECLSYFGVSETTGLTPDQVKRHLEKYGPNELPAEEGKSLWELVVEQFEDL
Mus_SERCA1	MEAAHSKSTEECLSYFGVSETTGLTPDQVKRHLEKYGPNELPAEEGKSLWELVVEQFEDL

	70 80 90 100 110 120
Tinamou_SERCA	LVRILLLAACISFVLAWFEEGEETITAFVEPFVILLILVANAIVGVWQERNAENAIEALK
Hummingbird_SERCA	LVRILLLAACISFVLAWFEEGEETITAFVEPFVILLILVANAIVGVWQERNAENAIEALK
Manakin_SERCA	LVRILLLAACISFVLAWFEEGEETITAFVEPFVILLILVANAIVGVWQERNAENAIEALK
Finch_SERCA1	LVRILLLAACISFVLAWFEEGEETITAFVEPFVILLILVANAIVGVWQERNAENAIEALK
Sparrow_SERCA	LVRILLLAACISFVLAWFEEGEETITAFVEPFVILLILVANAIVGVWQERNAENAIEALK
Starling_SERCA	LVRILLLAACISFVLAWFEEGEETITAFVEPFVILLILVANAIVGVWQERNAENAIEALK
Tibetan_ground-tit_SERCA	LVRILLLAACISFVLAWFEEGEETITAFVEPFVILLILVANAIVGVWQERNAENAIEALK
Ground-finch_SERCA	LVRILLLAACISFVLAWFEEGEETITAFVEPFVILLILVANAIVGVWQERNAENAIEALK
Downy_woodpecker_SERCA	LVRILLLAACISFVLAWFEEGEETITAFVEPFVILLILVANAIVGVWQERNAENAIEALK
Struthio_camelus_SERCA	LVRILLLAACISFVLAWFEEGEETITAFVEPFVILLILVANAIVGVWQERNAENAIEALK
Penguin_SERCA	LVRILLLAACISFVLAWFEEGEETITAFVEPFVILLILVANAIVGVWQERNAENAIEALK
Adelie_penguin_SERCA	LVRILLLAACISFVLAWFEEGEETITAFVEPFVILLILVANAIVGVWQERNAENAIEALK
Ruber_ruber_SERCA	LVRILLLAACISFVLAWFEEGEETITAFVEPFVILLILVANAIVGVWQERNAENAIEALK
Swift_SERCA	LVRILLLAACISFVLAWFEEGEETITAFVEPFVILLILVANAIVGVWQERNAENAIEALK
Falcon_SERCA	LVRILLLAACISFVLAWFEEGEETITAFVEPFVILLILVANAIVGVWQERNAENAIEALK
Japanese-quail_SERCA	LVRILLLAACISFVLAWFEEGEETITAFVEPFVILLILVANAIVGVWQERNAENAIEALK
White-tailed_tropicbird_SERCA	LVRILLLAACISFVLAWFEEGEETITAFVEPFVILLILVANAIVGVWQERNAENAIEALK
Ruff_SERCA	LVRILLLAACISFVLAWFEEGEETITAFVEPFVILLILVANAIVGVWQERNAENAIEALK
Turkey_SERCA	LVRILLLAACISFVLAWFEEGEETITAFVEPFVILLILVANAIVGVWQERNAENAIEALK
Little-egret_SERCA	LVRILLLAACISFVLAWFEEGEETITAFVEPFVILLILVANAIVGVWQERNAENAIEALK
Cuckoo_SERCA	LVRILLLAACISFVLAWFEEGEETITAFVEPFVILLILVANAIVGVWQERNAENAIEALK
Crowned-crane_SERCA	LVRILLLAACISFVLAWFEEGEETITAFVEPFVILLILVANAIVGVWQERNAENAIEALK
Chicken_SERCA1	LVRILLLAACISFVLAWFEEGEETITAFVEPFVILLILIANAVVGVWQERNAENAIEALK
Sifaka_SERCA1	LVRILLLAACISFVLAWFEEGEETITAFVEPFVILLILIANAIVGVWQERNAENAIEALK
Pongo_SERCA1	LVRILLLAACISFVLAWFEEGEETITAFVEPFVILLILIANAIVGVWQERNAENAIEALK
Human_SERCA1	LVRILLLAACISFVLAWFEEGEETITAFVEPFVILLILIANAIVGVWQERNAENAIEALK
Chimpanzee_SERCA1	LVRILLLAACISFVLAWFEEGEETITAFVEPFVILLILIANAIVGVWQERNAENAIEALK
Domestic_ferret_SERCA1	LVRILLLAACISFVLAWFEEGEETITAFVEPFVILLILIANAIVGVWQERNAENAIEALK
Dog_SERCA1	LVRILLLAACISFVLAWFEEGEETITAFVEPFVILLILIANAIVGVWQERNAENAIEALK
Panthera_SERCA1	LVRILLLAACISFVLAWFEEGEETITAFVEPFVILLILIANAIVGVWQERNAENAIEALK
Cat_SERCA1	LVRILLLAACISFVLAWFEEGEETITAFVEPFVILLILIANAIVGVWQERNAENAIEALK
Horseshoe-bat_SERCA1	LVRILLLAACISFVLAWFEEGEETVTAFVEPFVILLILIANAIVGVWQERNAENAIEALK
Vampire_bat_SERCA1	LVRILLLAACISFVLAWFEEGEETVTAFVEPFVILLILIANAIVGVWQERNAENAIEALK
Egyptian_rousette_SERCA1	LVRILLLAACISFVLAWFEEGEETVTAFVEPFVILLILVANAIVGVWQERNAENAIEALK
Philippine_tarsier_SERCA1	LVRILLLAACISFVLAWFEEGEETVTAFVEPFVILLILIANAIVGVWQERNAENAIEALK
Rabbit_SERCA1	LVRILLLAACISFVLAWFEEGEETITAFVEPFVILLILIANAIVGVWQERNAENAIEALK
American_pika_SERCA1	LVRILLLAACISFVLAWFEEGEETITAFVEPFVILLILIANAIVGVWQERNAENAIEALK
Thirteen-lined_ground_squirrel_SERCA1	LVRILLLAACISFVLAWFEEGEETITAFVEPFVILLILIANAIVGVWQERNAENAIEALK
Alpine_marmot_SERCA1	LVRILLLAACISFVLAWFEEGEETITAFVEPFVILLILIANAIVGVWQERNAENAIEALK
Chinchilla_SERCA1	LVRILLLAACISFVLAWFEEGEETITAFVEPFVILLILIANAIVGVWQERNAENAIEALK
Cape_elephant-shrew_SERCA1	LVRILLLAACISFVLAWFEEGEETITAFVEPFVILLILIANAIVGVWQERNAENAIEALK
Chinese_	LVRILLLAACISFVLAWFEEGEETITAFVEPFVILLILIANAIVGVWQERNAENAIEALK
Kangaroo_rat_SERCA1	LVRILLLAACISFVLAWFEEGEETITAFVEPFVILLILIANAIVGVWQERNAENAIEALK
Egyptian_jerboa_SERCA1	LVRILLLAACISFVLAWFEEGEETITAFVEPFVILLILIANAIVGVWQERNAENAIEALK
Mongolian_gerbil_SERCA1	LVRILLLAACISFVLAWFEEGEETVTAFVEPFVILLILIANAIVGVWQERNAENAIEALK
Prairie_vole_SERCA1	LVRILLLAACISFVLAWFEEGEETVTAFVEPFVILLILIANAIVGVWQERNAENAIEALK
Chinese	LVRILLLAACISFVLAWFEEGEETVTAFVEPFVILLILIANAIVGVWQERNAENAIEALK
Rattus_SERCA1	LVRILLLAACISFVLAWFEEGEETVTAFVEPFVILLILIANAIVGVWQERNAENAIEALK
Mus_SERCA1	LVRILLLAACISFVLAWFEEGEETVTAFVEPFVILLILIANAIVGVWQERNAENAIEALK

```
                                                   130       140       150        160       170
                                                     .         .         .          .         .
Tinamou_SERCA                              EYEPEMGKVYRQDRKSVQRIKARDIVPGDIVEVAVGDK.VPADIRITSIKSTTLRVDQSI
Hummingbird_SERCA                          EYEPEMGKVYRQDRESVQRIKARDIVPGDIVEVAVGDK.VPADIRLTSIKSTTLRVDQSI
Manakin_SERCA                              EYEPEMGKVYRQDRKSVQRIKARDIVPGDIVEVAVGDKFFPADIRITSIKSTTLRVDQSI
Finch_SERCA1                               EYEPEMGKVYRQDRKSVQRIKARDIVPGDIVEVAVGDK.VPADIRITSIKSTTLRVDQSI
Sparrow_SERCA                              EYEPEMGKVYRQDRKSVQRIKARDIVPGDIVEVAVGDK.VPADIRITSIKSTTLRVDQSI
Starling_SERCA                             EYEPEMGKVYRQDRKSVQRIKARDIVPGDIVEVAVGDK.VPADIRITSIKSTTLRVDQSI
Tibetan_ground-tit_SERCA                   EYEPEMGKVYRQDRKSVQRIKARDIVPGDIVEVAVGDK.VPADIRITSIKSTTLRVDQSI
Ground-finch_SERCA                         EYEPEMGKVYRQDRKSVQRIKARDIVPGDIVEVAVGDK.VLADIRITSIKSTTLRVDQSI
Downy_woodpecker_SERCA                     EYEPEMGKVYRQDRKSVQRIKARDIVPGDIVEVAGTAQ.LCSTYLITSIKSTTLRVDQSI
Struthio_camelus_SERCA                     EYEPEMGKVYRQDRKSVQRIKARDIVPGDIVEVAVNKL.LGIELRFKEIKSTTLRVDQSI
Penguin_SERCA                              EYEPEMGKVYRQDRKSVQRIKARDIVPGDIVEVAVGDK.VPADIRITSIKSTTLRVDQSI
Adelie_penguin_SERCA                       EYEPEMGKVYRQDRKSVQRIKARDIVPGDIVEVA.........VRITSIKSTTLRVDQSI
Ruber_ruber_SERCA                          EYEPEMGKVYRQDRKSVQRIKARDIVPGDIVEVAV............TSIKSTTLRVDQSI
Swift_SERCA                                EYEPEMGKVYRQDRKSVQRIKARDIVPGDIVEVAVGDK.GPADIRITSIKSTTLRVDQSI
Falcon_SERCA                               EYEPEMGKVYRQDRKSVQRIKARDIVPGDIVEVAVGDK.VPADIRITSIKSTTLRVDQSI
Japanese-quail_SERCA                       EYEPEMGKVYRQDRKSVQRIKARDIVPGDIVEVAVGDK.VPADIRITSIKSTTLRVDQSI
White-tailed_tropicbird_SERCA              EYEPEMGKVYRQDRKSVQRIKARDIVPGDIVEVAVGDK.V......TSIKSTTLRVDQSI
Ruff_SERCA                                 EYEPEMGKVYRQDRKSVQRIKARDIVPGDIVEVAVGDK.VPADIRITSIKSTTLRVDQSI
Turkey_SERCA                               EYEPEMGKVYRQDRKSVQRIKARDIVPGDIVEVAVGDK.VPADIRITSIKSTTLRVDQSI
Little-egret_SERCA                         EYEPEMGKVYRQDRKSVQRIKARDIVPGDIVEVAVGGK.VPADIRITSIKSTTLRVDQSI
Cuckoo_SERCA                               EYEPEMGKVYRQDRKSVQRIKARDIVPGDIVEVAVGDK.VPADIRITSIKSTTLRVDQSI
Crowned-crane_SERCA                        EYEPEMGKVYRQDRKSVQRIKARDIVPGDIVEVAVGDK.VPADIRITSIKSTTLRVDQSI
Chicken_SERCA1                             EYEPEMGKVYRADRKAVQRIKARDLVPGDIAEVAVGDK.VPADIRIISIKSTTLRVDQSI
Sifaka_SERCA1                              EYEPEMGKVYRADRKAVQRIKARDIVPGDIVEVAVGDK.VPADIRILSIKSTTLRVDQSI
Pongo_SERCA1                               EYEPEMGKVYRADRKSVQRIKARDIVPGDIVEVAVGDK.VPADIRILAIKSTTLRVDQSI
Human_SERCA1                               EYEPEMGKVYRADRKSVQRIKARDIVPGDIVEVAVGDK.VPADIRILAIKSTTLRVDQSI
Chimpanzee_SERCA1                          EYEPEMGKVYRADRKSVQRIKARDIVPGDIVEVAVGDK.VPADIRILAIKSTTLRVDQSI
Domestic_ferret_SERCA1                     EYEPEMGKVYRADRKSVQRIKARDIVPGDIVEVAVGDK.VPADIRILAIKSTTLRVDQSI
Dog_SERCA1                                 EYEPEMGKVYRADRKSVQRIKARDIVPGDIVEVAVGDK.VPADIRILSIKSTTLRVDQSI
Panthera_SERCA1                            EYEPEMGKVYRADRKTVQRIKARDIVPGDIVEVAVGDK.VPADIRILSIKSTTLRVDQSI
Cat_SERCA1                                 EYEPEMGKVYRADRKTVQRIKARDIVPGDIVEVAVGDK.VPADIRILSIKSTTLRVDQSI
Horseshoe-bat_SERCA1                       EYEPEMGKVYRADRKSVQRIKARDIVPGDIVEVAVGDK.VPADIRILSIKSTTLRVDQSI
Vampire_bat_SERCA1                         EYEPEMGKVYRADRKSVQRIKARDIVPGDIVEVAVGDK.VPADIRILSIKSTTLRVDQSI
Egyptian_rousette_SERCA1                   EYEPEMGKVYRADRKSVQRIKARDIVPGDIVEVAVGDK.VPADIRILSIKSTTLRVDQSI
Philippine_tarsier_SERCA1                  EYEPEMGKVYRADRKSVQRIKARDIVPGDIVEVAVGDK.VPADIRILAIKSTTLRVDQSI
Rabbit_SERCA1                              EYEPEMGKVYRADRKSVQRIKARDIVPGDIVEVAVGDK.VPADIRILSIKSTTLRVDQSI
American_pika_SERCA1                       EYEPEMGKVYRADRKSVQRIKARDIVPGDIVEVAVGDK.VPADIRILSIKSTTLRVDQSI
Thirteen-lined_ground_squirrel_SERCA1      EYEPEMGKVYRADRKSVQRIKARDIVPGDIVEVAVGDK.VPADIRILSIKSTTLRVDQSI
Alpine_marmot_SERCA1                       EYEPEMGKVYRADRKSVQRIKARDIVPGDIVEVAVGDK.VPADIRILSIKSTTLRVDQSI
Chinchilla_SERCA1                          EYEPEMGKVYRADRKSVQRIKAREMVPGDIVEVAVGDK.VPADIRILSIKSTTLRVDQSI
Cape_elephant-shrew_SERCA1                 EYEPEMGKVYRADRKSVQRIKARDIVPGDIVEVAVGDK.VPADIRILSIKSTTLRVDQSI
Chinese_                                   EYEPEMGKVYRADRKSVQRIKARDIVPGDIVEVAVGDK.VPADIRILSIKSTTLRVDQSI
Kangaroo_rat_SERCA1                        EYEPEMGKVYRADRKSVQRIKARDIVPGDIVEVAVGDK.VPADIRILSIKSTTLRVDQSI
Egyptian_jerboa_SERCA1                     EYEPEMGKVYRADRKTVQRIKARDIVPGDIVEVAVGDK.VPADIRILSIKSTTLRVDQSI
Mongolian_gerbil_SERCA1                    EYEPEMGKVYRADRKSVQRIKARDIVPGDIVEVAVGDK.VPADIRILSIKSTTLRVDQSI
Prairie_vole_SERCA1                        EYEPEMGKVYRADRKSVQRIKARDIVPGDIVEVAVGDK.VPADIRILSIKSTTLRVDQSI
Chinese                                    EYEPEMGKVYRADRKSVQRIKARDIVPGDIVEVAVGDK.VPADIRILSIKSTTLRVDQSI
Rattus_SERCA1                              EYEPEMGKVYRADRKSVQRIKARDIVPGDIVEVAVGDK.VPADIRILSIKSTTLRVDQSI
Mus_SERCA1                                 EYEPEMGKVYRADRKSVQRIKARDIVPGDIVEVAVGDK.VPADIRILSIKSTTLRVDQSI
```

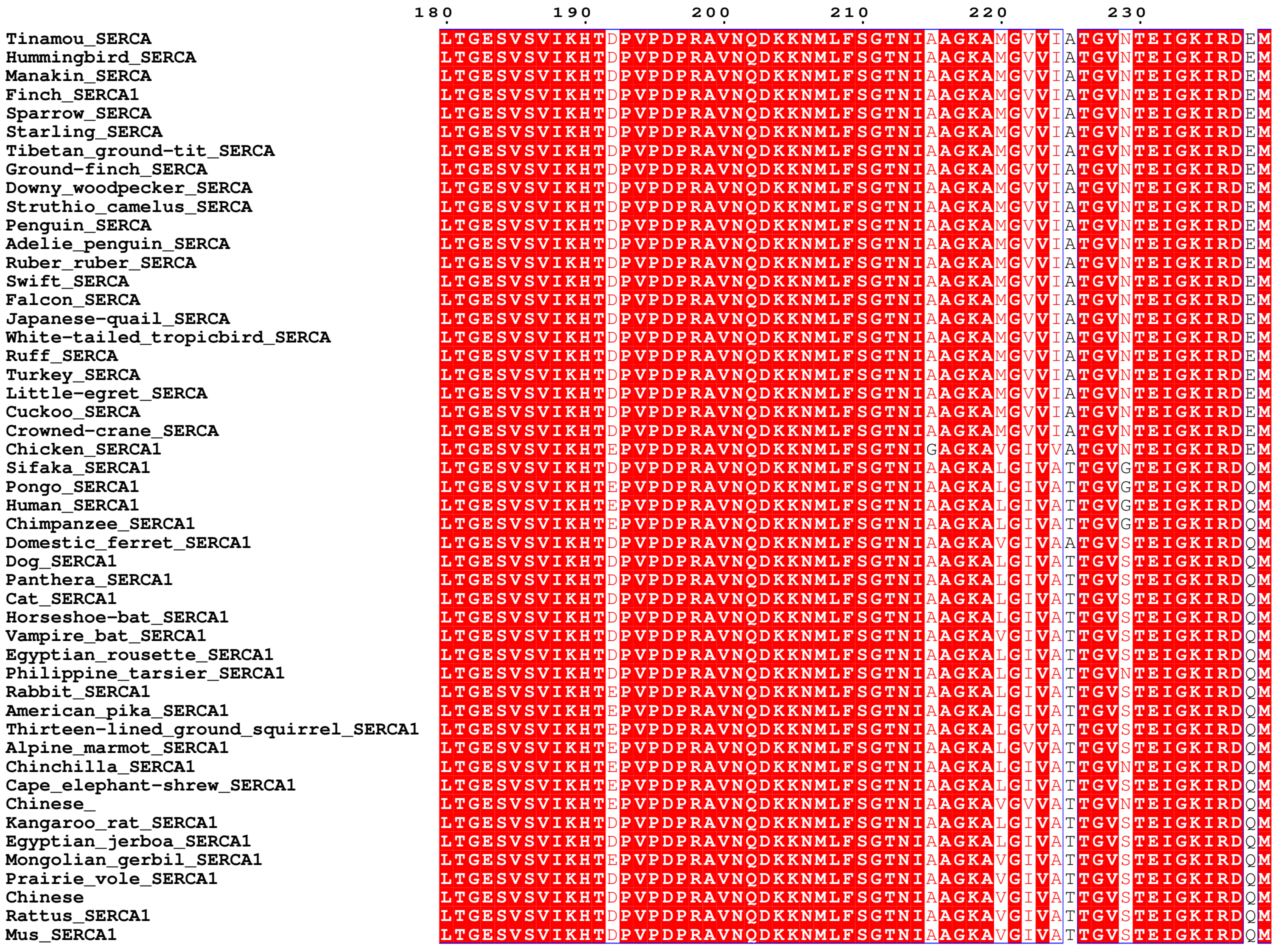
180 190 200 210 220 230
Tinamou_SERCA LTGESVSVIKHTDPVPDPRAVNQDKKNMLFSGTNIAAGKAMGVVIATGVNTEIGKIRDEM
Hummingbird_SERCA LTGESVSVIKHTDPVPDPRAVNQDKKNMLFSGTNIAAGKAMGVVIATGVNTEIGKIRDEM
Manakin_SERCA LTGESVSVIKHTDPVPDPRAVNQDKKNMLFSGTNIAAGKAMGVVIATGVNTEIGKIRDEM
Finch_SERCA1 LTGESVSVIKHTDPVPDPRAVNQDKKNMLFSGTNIAAGKAMGVVIATGVNTEIGKIRDEM
Sparrow_SERCA LTGESVSVIKHTDPVPDPRAVNQDKKNMLFSGTNIAAGKAMGVVIATGVNTEIGKIRDEM
Starling_SERCA LTGESVSVIKHTDPVPDPRAVNQDKKNMLFSGTNIAAGKAMGVVIATGVNTEIGKIRDEM
Tibetan_ground-tit_SERCA LTGESVSVIKHTDPVPDPRAVNQDKKNMLFSGTNIAAGKAMGVVIATGVNTEIGKIRDEM
Ground-finch_SERCA LTGESVSVIKHTDPVPDPRAVNQDKKNMLFSGTNIAAGKAMGVVIATGVNTEIGKIRDEM
Downy_woodpecker_SERCA LTGESVSVIKHTDPVPDPRAVNQDKKNMLFSGTNIAAGKAMGVVIATGVNTEIGKIRDEM
Struthio_camelus_SERCA LTGESVSVIKHTDPVPDPRAVNQDKKNMLFSGTNIAAGKAMGVVIATGVNTEIGKIRDEM
Penguin_SERCA LTGESVSVIKHTDPVPDPRAVNQDKKNMLFSGTNIAAGKAMGVVIATGVNTEIGKIRDEM
Adelie_penguin_SERCA LTGESVSVIKHTDPVPDPRAVNQDKKNMLFSGTNIAAGKAMGVVIATGVNTEIGKIRDEM
Ruber_ruber_SERCA LTGESVSVIKHTDPVPDPRAVNQDKKNMLFSGTNIAAGKAMGVVIATGVNTEIGKIRDEM
Swift_SERCA LTGESVSVIKHTDPVPDPRAVNQDKKNMLFSGTNIAAGKAMGVVIATGVNTEIGKIRDEM
Falcon_SERCA LTGESVSVIKHTDPVPDPRAVNQDKKNMLFSGTNIAAGKAMGVVIATGVNTEIGKIRDEM
Japanese-quail_SERCA LTGESVSVIKHTDPVPDPRAVNQDKKNMLFSGTNIAAGKAMGVVIATGVNTEIGKIRDEM
White-tailed_tropicbird_SERCA LTGESVSVIKHTDPVPDPRAVNQDKKNMLFSGTNIAAGKAMGVVIATGVNTEIGKIRDEM
Ruff_SERCA LTGESVSVIKHTDPVPDPRAVNQDKKNMLFSGTNIAAGKAMGVVIATGVNTEIGKIRDEM
Turkey_SERCA LTGESVSVIKHTDPVPDPRAVNQDKKNMLFSGTNIAAGKAMGVVIATGVNTEIGKIRDEM
Little-egret_SERCA LTGESVSVIKHTDPVPDPRAVNQDKKNMLFSGTNIAAGKAMGVVIATGVNTEIGKIRDEM
Cuckoo_SERCA LTGESVSVIKHTDPVPDPRAVNQDKKNMLFSGTNIAAGKAMGVVIATGVNTEIGKIRDEM
Crowned-crane_SERCA LTGESVSVIKHTDPVPDPRAVNQDKKNMLFSGTNIAAGKAMGVVIATGVNTEIGKIRDEM
Chicken_SERCA1 LTGESVSVIKHTEPVPDPRAVNQDKKNMLFSGTNIGAGKAVGIVVATGVNTEIGKIRDEM
Sifaka_SERCA1 LTGESVSVIKHTDPVPDPRAVNQDKKNMLFSGTNIAAGKALGIVATTGVGTEIGKIRDQM
Pongo_SERCA1 LTGESVSVIKHTEPVPDPRAVNQDKKNMLFSGTNIAAGKALGIVATTGVGTEIGKIRDQM
Human_SERCA1 LTGESVSVIKHTEPVPDPRAVNQDKKNMLFSGTNIAAGKALGIVATTGVGTEIGKIRDQM
Chimpanzee_SERCA1 LTGESVSVIKHTEPVPDPRAVNQDKKNMLFSGTNIAAGKALGIVATTGVGTEIGKIRDQM
Domestic_ferret_SERCA1 LTGESVSVIKHTDPVPDPRAVNQDKKNMLFSGTNIAAGKAVGIVAATGVSTEIGKIRDQM
Dog_SERCA1 LTGESVSVIKHTDPVPDPRAVNQDKKNMLFSGTNIAAGKALGIVATTGVSTEIGKIRDQM
Panthera_SERCA1 LTGESVSVIKHTDPVPDPRAVNQDKKNMLFSGTNIAAGKALGIVATTGVSTEIGKIRDQM
Cat_SERCA1 LTGESVSVIKHTDPVPDPRAVNQDKKNMLFSGTNIAAGKALGIVATTGVSTEIGKIRDQM
Horseshoe-bat_SERCA1 LTGESVSVIKHTDPVPDPRAVNQDKKNMLFSGTNIAAGKALGIVATTGVSTEIGKIRDQM
Vampire_bat_SERCA1 LTGESVSVIKHTDPVPDPRAVNQDKKNMLFSGTNIAAGKAVGIVATTGVSTEIGKIRDQM
Egyptian_rousette_SERCA1 LTGESVSVIKHTDPVPDPRAVNQDKKNMLFSGTNIAAGKALGIVATTGVSTEIGKIRDQM
Philippine_tarsier_SERCA1 LTGESVSVIKHTDPVPDPRAVNQDKKNMLFSGTNIAAGKALGIVATTGVNTEIGKIRDQM
Rabbit_SERCA1 LTGESVSVIKHTEPVPDPRAVNQDKKNMLFSGTNIAAGKALGIVATTGVSTEIGKIRDQM
American_pika_SERCA1 LTGESVSVIKHTEPVPDPRAVNQDKKNMLFSGTNIAAGKALGIVATTGVSTEIGKIRDQM
Thirteen-lined_ground_squirrel_SERCA1 LTGESVSVIKHTEPVPDPRAVNQDKKNMLFSGTNIAAGKALGVVATTGVSTEIGKIRDQM
Alpine_marmot_SERCA1 LTGESVSVIKHTEPVPDPRAVNQDKKNMLFSGTNIAAGKALGVVATTGVSTEIGKIRDQM
Chinchilla_SERCA1 LTGESVSVIKHTEPVPDPRAVNQDKKNMLFSGTNIAAGKALGIVATTGVNTEIGKIRDQM
Cape_elephant-shrew_SERCA1 LTGESVSVIKHTEPVPDPRAVNQDKKNMLFSGTNIAAGKALGIVATTGVSTEIGKIRDQM
Chinese_ LTGESVSVIKHTEPVPDPRAVNQDKKNMLFSGTNIAAGKAVGVVATTGVNTEIGKIRDQM
Kangaroo_rat_SERCA1 LTGESVSVIKHTDPVPDPRAVNQDKKNMLFSGTNIAAGKALGIVATTGVSTEIGKIRDQM
Egyptian_jerboa_SERCA1 LTGESVSVIKHTDPVPDPRAVNQDKKNMLFSGTNIAAGKALGIVATTGVSTEIGKIRDQM
Mongolian_gerbil_SERCA1 LTGESVSVIKHTEPVPDPRAVNQDKKNMLFSGTNIAAGKAVGIVATTGVSTEIGKIRDQM
Prairie_vole_SERCA1 LTGESVSVIKHTDPVPDPRAVNQDKKNMLFSGTNIAAGKAVGIVATTGVSTEIGKIRDQM
Chinese LTGESVSVIKHTDPVPDPRAVNQDKKNMLFSGTNIAAGKAVGIVATTGVSTEIGKIRDQM
Rattus_SERCA1 LTGESVSVIKHTDPVPDPRAVNQDKKNMLFSGTNIAAGKAVGIVATTGVSTEIGKIRDQM
Mus_SERCA1 LTGESVSVIKHTDPVPDPRAVNQDKKNMLFSGTNIAAGKAVGIVATTGVSTEIGKIRDQM

```
                                          240       250       260       270       280       290
Tinamou_SERCA                            VATEQERTPLQQKLDEFGEQLSKVISLICIAVWIINIGHFNDPVHGGSWIRGAIYYFKIA
Hummingbird_SERCA                        VATEQERTPLQQKLDEFGEQLSKVISLICIAVWIINIGHFNDPVHGGSWIRGAIYYFKIA
Manakin_SERCA                            VATEQERTPLQQKLDEFGEQLSKVISLICIAVWIINIGHFNDPVHGGSWIRGAIYYFKIA
Finch_SERCA1                             VATEQERTPLQQKLDEFGEQLSKVISLICIAVWIINIGHFNDPVHGGSWIRGAIYYFKIA
Sparrow_SERCA                            VATEQERTPLQQKLDEFGEQLSKVISLICIAVWIINIGHFNDPVHGGSWIRGAIYYFKIA
Starling_SERCA                           VATEQERTPLQQKLDEFGEQLSKVISLICIAVWIINIGHFNDPVHGGSWIRGAIYYFKIA
Tibetan_ground-tit_SERCA                 VATEQERTPLQQKLDEFGEQLSKVISLICIAVWIINIGHFNDPVHGGSWIRGAIYYFKIA
Ground-finch_SERCA                       VATEQERTPLQQKLDEFGEQLSKVISLICIAVWIINIGHFNDPVHGGSWIRGAIYYFKIA
Downy_woodpecker_SERCA                   VATEQERTPLQQKLDEFGEQLSKVISLICIAVWIINIGHFNDPVHGGSWIRGAIYYFKIA
Struthio_camelus_SERCA                   VATEQERTPLQQKLDEFGEQLSKVISLICIAVWIINIGHFNDPVHGGSWIRGAIYYFKIA
Penguin_SERCA                            VATEQERTPLQQKLDEFGEQLSKVISLICIAVWIINIGHFNDPVHGGSWIRGAIYYFKIA
Adelie_penguin_SERCA                     VATEQERTPLQQKLDEFGEQLSKVISLICIAVWIINIGHFNDPVHGGSWIRGAIYYFKIA
Ruber_ruber_SERCA                        VATEQERTPLQQKLDEFGEQLSKVISLICIAVWIINIGHFNDPVHGGSWIRGAIYYFKIA
Swift_SERCA                              VATEQERTPLQQKLDEFGEQLSKVISLICIAVWIINIGHFNDPVHGGSWIRGAIYYFKIA
Falcon_SERCA                             VATEQERTPLQQKLDEFGEQLSKVISLICIAVWIINIGHFNDPVHGGSWIRGAIYYFKIA
Japanese-quail_SERCA                     VATEQERTPLQQKLDEFGEQLSKVISLICIAVWIINIGHFNDPVHGGSWIRGAIYYFKIA
White-tailed_tropicbird_SERCA            VATEQERTPLQQKLDEFGEQLSKVISLICIAVWIINIGHFNDPVHGGSWIRGAIYYFKIA
Ruff_SERCA                               VATEQERTPLQQKLDEFGEQLSKVISLICIAVWIINIGHFNDPVHGGSWIRGAIYYFKIA
Turkey_SERCA                             VATEQERTPLQQKLDEFGEQLSKVISLICIAVWIINIGHFNDPVHGGSWIRGAIYYFKIA
Little-egret_SERCA                       VATEQERTPLQQKLDEFGEQLSKVISLICIAVWIINIGHFNDPVHGGSWIRGAIYYFKIA
Cuckoo_SERCA                             VATEQERTPLQQKLDEFGEQLSKVISLICIAVWIINIGHFNDPVHGGSWIRGAIYYFKIA
Crowned-crane_SERCA                      VATEQERTPLQQKLDEFGEQLSKVISLICIAVWIINIGHFNDPVHGGSWIRGAIYYFKIA
Chicken_SERCA1                           AATEQDKTPLQQKLDEFGEQLSKVISLICVAVWLINIGHFNDPVHGGSWIRGAIYYFKIA
Sifaka_SERCA1                            AATEQEKTPLQQKLDEFGEQLSKVISLICVAVWLINIGHFNDPVHGGSWFRGAIYYFKIA
Pongo_SERCA1                             AATEQDKTPLQQKLDEFGEQLSKVISLICVAVWLINIGHFNDPVHGGSWFRGAIYYFKIA
Human_SERCA1                             AATEQDKTPLQQKLDEFGEQLSKVISLICVAVWLINIGHFNDPVHGGSWFRGAIYYFKIA
Chimpanzee_SERCA1                        AATEQDKTPLQQKLDEFGEQLSKVISLICVAVWLINIGHFNDPVHGGSWFRGAIYYFKIA
Domestic_ferret_SERCA1                   AATEQDKTPLQQKLDEFGEQLSKVISLICVAVWLINIGHFNDPVHGGSWFRGAIYYFKIA
Dog_SERCA1                               AATEQDKTPLQQKLDEFGEQLSKVISLICVAVWLINIGHFNDPVHGGSWIRGAIYYFKIA
Panthera_SERCA1                          AATEQDKTPLQQKLDEFGEQLSKVISLICVAVWLINIGHFNDPVHGGSWLRGAIYYFKIA
Cat_SERCA1                               AATEQDKTPLQQKLDEFGEQLSKVISLICVAVWLINIGHFNDPVHGGSWLRGAIYYFKIA
Horseshoe-bat_SERCA1                     AATEQDKTPLQQKLDEFGEQLSKVISLICVAVWLINIGHFNDPVHGGSWFRGAIYYFKIA
Vampire_bat_SERCA1                       AATEQDKTPLQQKLDEFGEQLSKVISLICVAVWLINIGHFNDPVHGGSWFRGAIYYFKIA
Egyptian_rousette_SERCA1                 AATEQDKTPLQQKLDEFGEQLSKVISLICVAVWLINIGHFNDPVHGGSWFRGAIYYFKIA
Philippine_tarsier_SERCA1                AATEQEKTPLQQKLDEFGEQLSKVISLICVAVWLINIGHFNDPVHGGSWFRGAIYYFKIA
Rabbit_SERCA1                            AATEQDKTPLQQKLDEFGEQLSKVISLICVAVWLINIGHFNDPVHGGSWIRGAIYYFKIA
American_pika_SERCA1                     AATEQDKTPLQQKLDEFGEQLSKVISLICVAVWLINIGHFNDPVHGGSWIRGAIYYFKIA
Thirteen-lined_ground_squirrel_SERCA1    AATEQDKTPLQQKLDEFGEQLSKVISLICVAVWLINIGHFNDPVHGGSWFRGAIYYFKIA
Alpine_marmot_SERCA1                     AATEQDKTPLQQKLDEFGEQLSKVISLICVAVWLINIGHFNDPVHGGSWFRGAIYYFKIA
Chinchilla_SERCA1                        AATEQDKTPLQQKLDEFGEQLSKVISLICVAVWLINIGHFNDPVHGGSWFRGAIYYFKIA
Cape_elephant-shrew_SERCA1               AATEQEKTPLQQKLDEFGEQLSKVISLICVAVWLINIGHFNDPVHGGSWFRGAIYYFKIA
Chinese_                                 AATEQDKTPLQQKLDEFGEQLSKVISLICVAVWLINIGHFNDPVHGGSWFRGAIYYFKIA
Kangaroo_rat_SERCA1                      AATEQDKTPLQQKLDEFGEQLSKVISLICVAVWLINIGHFNDPVHGGSWFRGAIYYFKIA
Egyptian_jerboa_SERCA1                   AATEQDKTPLQQKLDEFGEQLSKVISLICVAVWLINIGHFNDPVHGGSWFRGAIYYFKIA
Mongolian_gerbil_SERCA1                  AATEQDKTPLQQKLDEFGEQLSKVISLICVAVWLINIGHFNDPVHGGSWFRGAIYYFKIA
Prairie_vole_SERCA1                      AATEQDKTPLQQKLDEFGEQLSKVISLICVAVWLINIGHFNDPVHGGSWFRGAIYYFKIA
Chinese                                  AATEQDKTPLQQKLDEFGEQLSKVISLICVAVWLINIGHFNDPVHGGSWFRGAIYYFKIA
Rattus_SERCA1                            AATEQDKTPLQQKLDEFGEQLSKVISLICVAVWLINIGHFNDPVHGGSWFRGAIYYFKIA
Mus_SERCA1                               AATEQDKTPLQQKLDEFGEQLSKVISLICVAVWLINIGHFNDPVHGGSWFRGAIYYFKIA
```

	300 · 310 · 320 · 330 · 340 · 350
Tinamou_SERCA	VALAVAAIPEGLPAVITTCLALGTRRMAKKNAIVRSLPSVETLGCTSVICSDKTGTLTTN
Hummingbird_SERCA	VALAVAAIPEGLPAVITTCLALGTRRMAKKNAIVRSLPSVETLGCTSVICSDKTGTLTTN
Manakin_SERCA	VALAVAAIPEGLPAVITTCLALGTRRMAKKNAIVRSLPSVETLGCTSVICSDKTGTLTTN
Finch_SERCA1	VALAVAAIPEGLPAVITTCLALGTRRMAKKNAIVRSLPSVETLGCTSVICSDKTGTLTTN
Sparrow_SERCA	VALAVAAIPEGLPAVITTCLALGTRRMAKKNAIVRSLPSVETLGCTSVICSDKTGTLTTN
Starling_SERCA	VALAVAAIPEGLPAVITTCLALGTRRMAKKNAIVRSLPSVETLGCTSVICSDKTGTLTTN
Tibetan_ground-tit_SERCA	VALAVAAIPEGLPAVITTCLALGTRRMAKKNAIVRSLPSVETLGCTSVICSDKTGTLTTN
Ground-finch_SERCA	VALAVAAIPEGLPAVITTCLALGTRRMAKKNAIVRSLPSVETLGCTSVICSDKTGTLTTN
Downy_woodpecker_SERCA	VALAVAAIPEGLPAVITTCLALGTRRMAKKNAIVRSLPSVETLGCTSVICSDKTGTLTTN
Struthio_camelus_SERCA	VALAVAAIPEGLPAVITTCLALGTRRMAKKNAIVRSLPSVETLGCTSVICSDKTGTLTTN
Penguin_SERCA	VALAVAAIPEGLPAVITTCLALGTRRMAKKNAIVRSLPSVETLGCTSVICSDKTGTLTTN
Adelie_penguin_SERCA	VALAVAAIPEGLPAVITTCLALGTRRMAKKNAIVRSLPSVETLGCTSVICSDKTGTLTTN
Ruber_ruber_SERCA	VALAVAAIPEGLPAVITTCLALGTRRMAKKNAIVRSLPSVETLGCTSVICSDKTGTLTTN
Swift_SERCA	VALAVAAIPEGLPAVITTCLALGTRRMAKKNAIVRSLPSVETLGCTSVICSDKTGTLTTN
Falcon_SERCA	VALAVAAIPEGLPAVITTCLALGTRRMAKKNAIVRSLPSVETLGCTSVICSDKTGTLTTN
Japanese-quail_SERCA	VALAVAAIPEGLPAVITTCLALGTRRMAKKNAIVRSLPSVETLGCTSVICSDKTGTLTTN
White-tailed_tropicbird_SERCA	VALAVAAIPEGLPAVITTCLALGTRRMAKKNAIVRSLPSVETLGCTSVICSDKTGTLTTN
Ruff_SERCA	VALAVAAIPEGLPAVITTCLALGTRRMAKKNAIVRSLPSVETLGCTSVICSDKTGTLTTN
Turkey_SERCA	VALAVAAIPEGLPAVITTCLALGTRRMAKKNAIVRSLPSVETLGCTSVICSDKTGTLTTN
Little-egret_SERCA	VALAVAAIPEGLPAVITTCLALGTRRMAKKNAIVRSLPSVETLGCTSVICSDKTGTLTTN
Cuckoo_SERCA	VALAVAAIPEGLPAVITTCLALGTRRMAKKNAIVRSLPSVETLGCTSVICSDKTGTLTTN
Crowned-crane_SERCA	VALAVAAIPEGLPAVITTCLALGTRRMAKKNAIVRSLPSVETLGCTSVICSDKTGTLTTN
Chicken_SERCA1	VALAVAAIPEGLPAVITTCLALGTRRMAKKNAIVRSLPSVETLGCTSVICSDKTGTLTTN
Sifaka_SERCA1	VALAVAAIPEGLPAVITTCLALGTRRMAKKNAIVRSLPSVETLGCTSVICSDKTGTLTTN
Pongo_SERCA1	VALAVAAIPEGLPAVITTCLALGTRRMAKKNAIVRSLPSVETLGCTSVICSDKTGTLTTN
Human_SERCA1	VALAVAAIPEGLPAVITTCLALGTRRMAKKNAIVRSLPSVETLGCTSVICSDKTGTLTTN
Chimpanzee_SERCA1	VALAVAAIPEGLPAVITTCLALGTRRMAKKNAIVRSLPSVETLGCTSVICSDKTGTLTTN
Domestic_ferret_SERCA1	VALAVAAIPEGLPAVITTCLALGTRRMAKKNAIVRSLPSVETLGCTSVICSDKTGTLTTN
Dog_SERCA1	VALAVAAIPEGLPAVITTCLALGTRRMAKKNAIVRSLPSVETLGCTSVICSDKTGTLTTN
Panthera_SERCA1	VALAVAAIPEGLPAVITTCLALGTRRMAKKNAIVRSLPSVETLGCTSVICSDKTGTLTTN
Cat_SERCA1	VALAVAAIPEGLPAVITTCLALGTRRMAKKNAIVRSLPSVETLGCTSVICSDKTGTLTTN
Horseshoe-bat_SERCA1	VALAVAAIPEGLPAVITTCLALGTRRMAKKNAIVRSLPSVETLGCTSVICSDKTGTLTTN
Vampire_bat_SERCA1	VALAVAAIPEGLPAVITTCLALGTRRMAKKNAIVRSLPSVETLGCTSVICSDKTGTLTTN
Egyptian_rousette_SERCA1	VALAVAAIPEGLPAVITTCLALGTRRMAKKNAIVRSLPSVETLGCTSVICSDKTGTLTTN
Philippine_tarsier_SERCA1	VALAVAAIPEGLPAVITTCLALGTRRMAKKNAIVRSLPSVETLGCTSVICSDKTGTLTTN
Rabbit_SERCA1	VALAVAAIPEGLPAVITTCLALGTRRMAKKNAIVRSLPSVETLGCTSVICSDKTGTLTTN
American_pika_SERCA1	VALAVAAIPEGLPAVITTCLALGTRRMAKKNAIVRSLPSVETLGCTSVICSDKTGTLTTN
Thirteen-lined_ground_squirrel_SERCA1	VALAVAAIPEGLPAVITTCLALGTRRMAKKNAIVRSLPSVETLGCTSVICSDKTGTLTTN
Alpine_marmot_SERCA1	VALAVAAIPEGLPAVITTCLALGTRRMAKKNAIVRSLPSVETLGCTSVICSDKTGTLTTN
Chinchilla_SERCA1	VALAVAAIPEGLPAVITTCLALGTRRMAKKNAIVRSLPSVETLGCTSVICSDKTGTLTTN
Cape_elephant-shrew_SERCA1	VALAVAAIPEGLPAVITTCLALGTRRMAKKNAIVRSLPSVETLGCTSVICSDKTGTLTTN
Chinese_	VALAVAAIPEGLPAVITTCLALGTRRMAKKNAIVRSLPSVETLGCTSVICSDKTGTLTTN
Kangaroo_rat_SERCA1	VALAVAAIPEGLPAVITTCLALGTRRMAKKNAIVRSLPSVETLGCTSVICSDKTGTLTTN
Egyptian_jerboa_SERCA1	VALAVAAIPEGLPAVITTCLALGTRRMAKKNAIVRSLPSVETLGCTSVICSDKTGTLTTN
Mongolian_gerbil_SERCA1	VALAVAAIPEGLPAVITTCLALGTRRMAKKNAIVRSLPSVETLGCTSVICSDKTGTLTTN
Prairie_vole_SERCA1	VALAVAAIPEGLPAVITTCLALGTRRMAKKNAIVRSLPSVETLGCTSVICSDKTGTLTTN
Chinese	VALAVAAIPEGLPAVITTCLALGTRRMAKKNAIVRSLPSVETLGCTSVICSDKTGTLTTN
Rattus_SERCA1	VALAVAAIPEGLPAVITTCLALGTRRMAKKNAIVRSLPSVETLGCTSVICSDKTGTLTTN
Mus_SERCA1	VALAVAAIPEGLPAVITTCLALGTRRMAKKNAIVRSLPSVETLGCTSVICSDKTGTLTTN

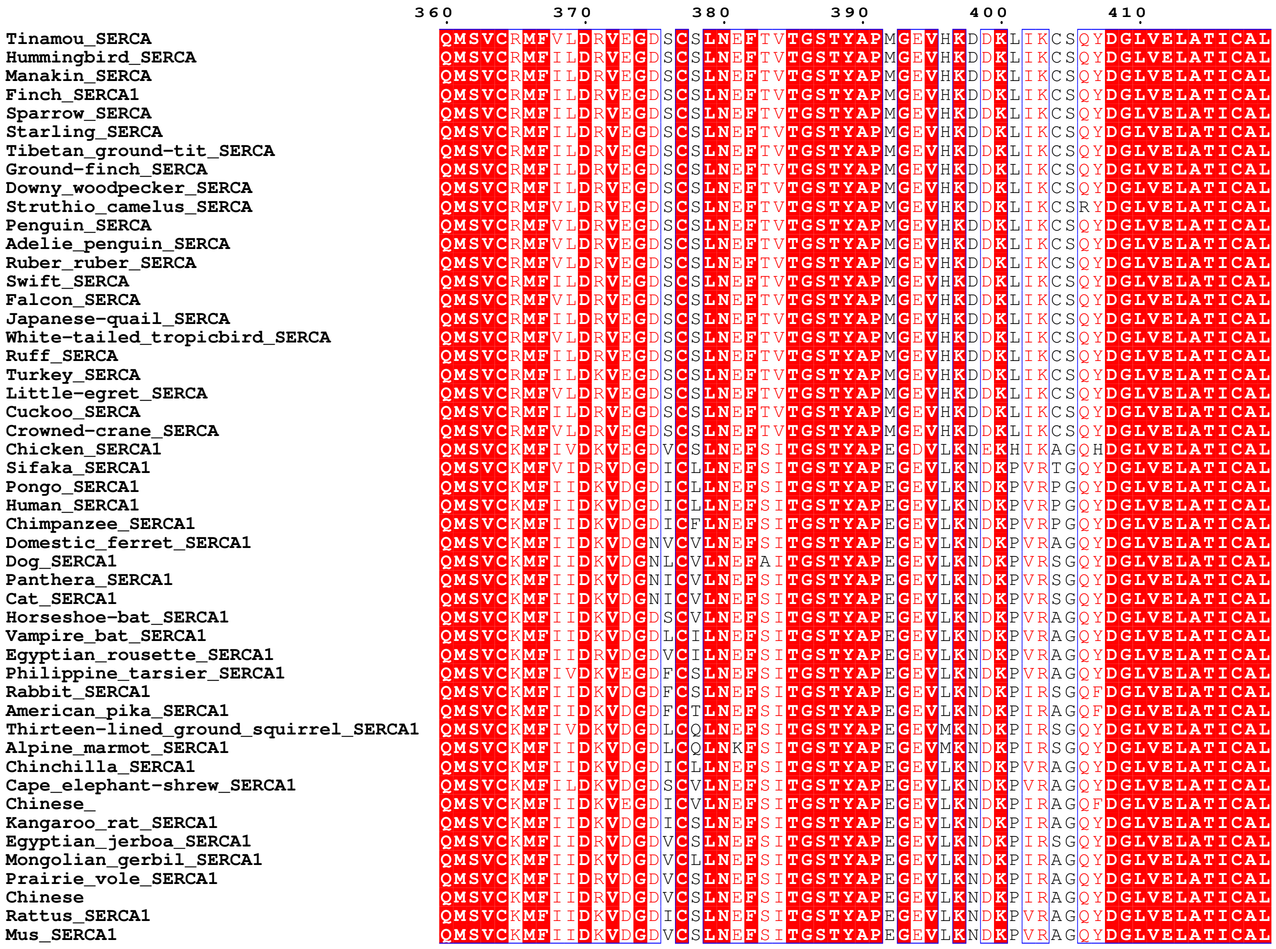
360 370 380 390 400 410
Tinamou_SERCA QMSVCRMFVLDRVEGDSCSLNEFTVTGSTYAPMGEVHKDDKLIKCSQYDGLVELATICAL
Hummingbird_SERCA QMSVCRMFILDRVEGDSCSLNEFTVTGSTYAPMGEVHKDDKLIKCSQYDGLVELATICAL
Manakin_SERCA QMSVCRMFILDRVEGDSCSLNEFTVTGSTYAPMGEVHKDDKLIKCSQYDGLVELATICAL
Finch_SERCA1 QMSVCRMFILDRVEGDSCSLNEFTVTGSTYAPMGEVHKDDKLIKCSQYDGLVELATICAL
Sparrow_SERCA QMSVCRMFILDRVEGDSCSLNEFTVTGSTYAPMGEVHKDDKLIKCSQYDGLVELATICAL
Starling_SERCA QMSVCRMFILDRVEGDSCSLNEFTVTGSTYAPMGEVHKDDKLIKCSQYDGLVELATICAL
Tibetan_ground-tit_SERCA QMSVCRMFILDRVEGDSCSLNEFTVTGSTYAPMGEVHKDDKLIKCSQYDGLVELATICAL
Ground-finch_SERCA QMSVCRMFILDRVEGDSCSLNEFTVTGSTYAPMGEVHKDDKLIKCSQYDGLVELATICAL
Downy_woodpecker_SERCA QMSVCRMFILDRVEGDSCSLNEFTVTGSTYAPMGEVHKDDKLIKCSQYDGLVELATICAL
Struthio_camelus_SERCA QMSVCRMFILDRVEGDSCSLNEFTVTGSTYAPMGEVHKDDKLIKCSRYDGLVELATICAL
Penguin_SERCA QMSVCRMFVLDRVEGDSCSLNEFTVTGSTYAPMGEVHKDDKLIKCSQYDGLVELATICAL
Adelie_penguin_SERCA QMSVCRMFVLDRVEGDSCSLNEFTVTGSTYAPMGEVHKDDKLIKCSQYDGLVELATICAL
Ruber_ruber_SERCA QMSVCRMFVLDRVEGDSCSLNEFTVTGSTYAPMGEVHKDDKLIKCSQYDGLVELATICAL
Swift_SERCA QMSVCRMFILDRVEGDSCSLNEFTVTGSTYAPMGEVHKDDKLIKCSQYDGLVELATICAL
Falcon_SERCA QMSVCRMFVLDRVEGDSCSLNEFTVTGSTYAPMGEVHKDDKLIKCSQYDGLVELATICAL
Japanese-quail_SERCA QMSVCRMFILDRVEGDSCSLNEFTVTGSTYAPMGEVHKDDKLIKCSQYDGLVELATICAL
White-tailed_tropicbird_SERCA QMSVCRMFILDRVEGDSCSLNEFTVTGSTYAPMGEVHKDDKLIKCSQYDGLVELATICAL
Ruff_SERCA QMSVCRMFILDRVEGDSCSLNEFTVTGSTYAPMGEVHKDDKLIKCSQYDGLVELATICAL
Turkey_SERCA QMSVCRMFILDKVEGDSCSLNEFTVTGSTYAPMGEVHKDDKLIKCSQYDGLVELATICAL
Little-egret_SERCA QMSVCRMFVLDRVEGDSCSLNEFTVTGSTYAPMGEVHKDDKLIKCSQYDGLVELATICAL
Cuckoo_SERCA QMSVCRMFILDRVEGDSCSLNEFTVTGSTYAPMGEVHKDDKLIKCSQYDGLVELATICAL
Crowned-crane_SERCA QMSVCRMFVLDRVEGDSCSLNEFTVTGSTYAPMGEVHKDDKLIKCSQYDGLVELATICAL
Chicken_SERCA1 QMSVCKMFIVDKVEGDVCSLNEFSITGSTYAPEGDVLKNEKHIKAGQHDGLVELATICAL
Sifaka_SERCA1 QMSVCKMFVIDRVDGDICLLNEFSITGSTYAPEGEVLKNDKPVRTGQYDGLVELATICAL
Pongo_SERCA1 QMSVCKMFIIDKVDGDICLLNEFSITGSTYAPEGEVLKNDKPVRPGQYDGLVELATICAL
Human_SERCA1 QMSVCKMFIIDKVDGDICLLNEFSITGSTYAPEGEVLKNDKPVRPGQYDGLVELATICAL
Chimpanzee_SERCA1 QMSVCKMFIIDKVDGDICFLNEFSITGSTYAPEGEVLKNDKPVRPGQYDGLVELATICAL
Domestic_ferret_SERCA1 QMSVCKMFIIDKVDGNVCVLNEFSITGSTYAPEGEVLKNDKPVRAGQYDGLVELATICAL
Dog_SERCA1 QMSVCKMFIIDKVDGNLCVLNEFAITGSTYAPEGEVLKNDKPVRSGQYDGLVELATICAL
Panthera_SERCA1 QMSVCKMFIIDKVDGNICVLNEFSITGSTYAPEGEVLKNDKPVRSGQYDGLVELATICAL
Cat_SERCA1 QMSVCKMFIIDKVDGNICVLNEFSITGSTYAPEGEVLKNDKPVRSGQYDGLVELATICAL
Horseshoe-bat_SERCA1 QMSVCKMFIIDKVDGDSCVLNEFSITGSTYAPEGEVLKNDKPVRAGQYDGLVELATICAL
Vampire_bat_SERCA1 QMSVCKMFIIDKVDGDLCILNEFSITGSTYAPEGEVLKNDKPVRAGQYDGLVELATICAL
Egyptian_rousette_SERCA1 QMSVCKMFIIDRVDGDVCILNEFSITGSTYAPEGEVLKNDKPVRAGQYDGLVELATICAL
Philippine_tarsier_SERCA1 QMSVCKMFIVDKVEGDFCSLNEFSITGSTYAPEGEVLKNDKPVRAGQYDGLVELATICAL
Rabbit_SERCA1 QMSVCKMFIIDKVDGDFCSLNEFSITGSTYAPEGEVLKNDKPIRSGQFDGLVELATICAL
American_pika_SERCA1 QMSVCKMFIIDKVDGDFCTLNEFSITGSTYAPEGEVLKNDKPIRAGQFDGLVELATICAL
Thirteen-lined_ground_squirrel_SERCA1 QMSVCKMFIVDKVDGDLCQLNEFSITGSTYAPEGEVMKNDKPIRSGQYDGLVELATICAL
Alpine_marmot_SERCA1 QMSVCKMFIIDKVDGDLCQLNKFSITGSTYAPEGEVLKNDKPIRSGQYDGLVELATICAL
Chinchilla_SERCA1 QMSVCKMFIIDKVDGDICLLNEFSITGSTYAPEGEVLKNDKPVRAGQYDGLVELATICAL
Cape_elephant-shrew_SERCA1 QMSVCKMFILDKVDGDSCVLNEFSITGSTYAPEGEVLKNDKPVRAGQYDGLVELATICAL
Chinese_ QMSVCKMFIIDKVEGDICVLNEFSITGSTYAPEGEVLKNDKPIRAGQFDGLVELATICAL
Kangaroo_rat_SERCA1 QMSVCKMFIIDKVDGDICSLNEFSITGSTYAPEGEVLKNDKPIRAGQYDGLVELATICAL
Egyptian_jerboa_SERCA1 QMSVCKMFIIDRVDGDVCSLNEFSITGSTYAPEGEVLKNDKPIRSGQYDGLVELATICAL
Mongolian_gerbil_SERCA1 QMSVCKMFIIDKVDGDVCLLNEFSITGSTYAPEGEVLKNDKPIRAGQYDGLVELATICAL
Prairie_vole_SERCA1 QMSVCKMFIIDRVDGDVCSLNEFSITGSTYAPEGEVLKNDKPIRAGQYDGLVELATICAL
Chinese QMSVCKMFIIDRVDGDVCSLNEFSITGSTYAPEGEVLKNDKPIRAGQYDGLVELATICAL
Rattus_SERCA1 QMSVCKMFIIDKVDGDICSLNEFSITGSTYAPEGEVLKNDKPVRAGQYDGLVELATICAL
Mus_SERCA1 QMSVCKMFIIDKVDGDVCSLNEFSITGSTYAPEGEVLKNDKPVRAGQYDGLVELATICAL

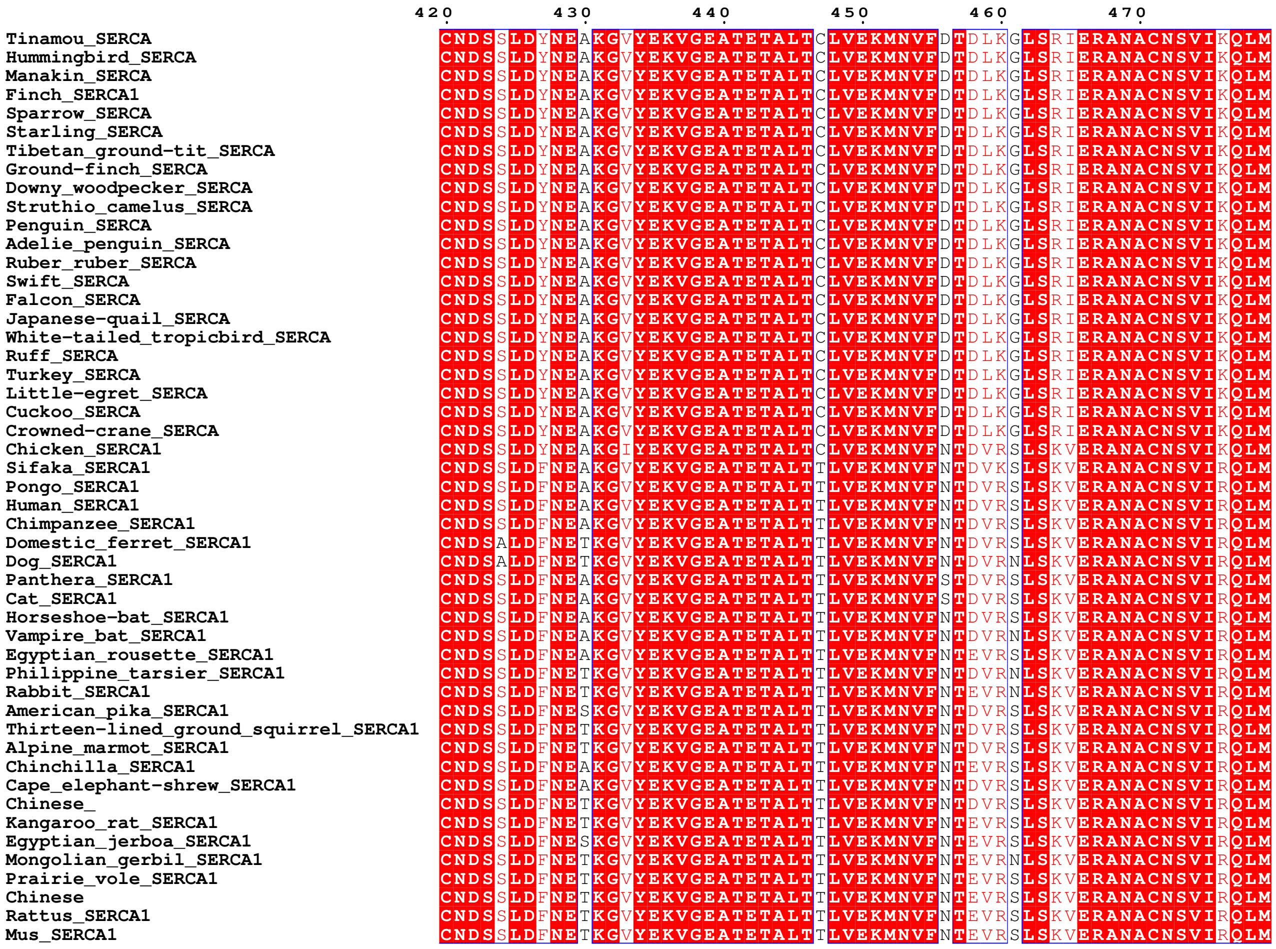
420 430 440 450 460 470
Tinamou_SERCA CNDSSLDYNEAKGVYEKVGEATETALTCLVEKMNVFDTDLKGLSRIERANACNSVIKQLM
Hummingbird_SERCA CNDSSLDYNEAKGVYEKVGEATETALTCLVEKMNVFDTDLKGLSRIERANACNSVIKQLM
Manakin_SERCA CNDSSLDYNEAKGVYEKVGEATETALTCLVEKMNVFDTDLKGLSRIERANACNSVIKQLM
Finch_SERCA1 CNDSSLDYNEAKGVYEKVGEATETALTCLVEKMNVFDTDLKGLSRIERANACNSVIKQLM
Sparrow_SERCA CNDSSLDYNEAKGVYEKVGEATETALTCLVEKMNVFDTDLKGLSRIERANACNSVIKQLM
Starling_SERCA CNDSSLDYNEAKGVYEKVGEATETALTCLVEKMNVFDTDLKGLSRIERANACNSVIKQLM
Tibetan_ground-tit_SERCA CNDSSLDYNEAKGVYEKVGEATETALTCLVEKMNVFDTDLKGLSRIERANACNSVIKQLM
Ground-finch_SERCA CNDSSLDYNEAKGVYEKVGEATETALTCLVEKMNVFDTDLKGLSRIERANACNSVIKQLM
Downy_woodpecker_SERCA CNDSSLDYNEAKGVYEKVGEATETALTCLVEKMNVFDTDLKGLSRIERANACNSVIKQLM
Struthio_camelus_SERCA CNDSSLDYNEAKGVYEKVGEATETALTCLVEKMNVFDTDLKGLSRIERANACNSVIKQLM
Penguin_SERCA CNDSSLDYNEAKGVYEKVGEATETALTCLVEKMNVFDTDLKGLSRIERANACNSVIKQLM
Adelie_penguin_SERCA CNDSSLDYNEAKGVYEKVGEATETALTCLVEKMNVFDTDLKGLSRIERANACNSVIKQLM
Ruber_ruber_SERCA CNDSSLDYNEAKGVYEKVGEATETALTCLVEKMNVFDTDLKGLSRIERANACNSVIKQLM
Swift_SERCA CNDSSLDYNEAKGVYEKVGEATETALTCLVEKMNVFDTDLKGLSRIERANACNSVIKQLM
Falcon_SERCA CNDSSLDYNEAKGVYEKVGEATETALTCLVEKMNVFDTDLKGLSRIERANACNSVIKQLM
Japanese-quail_SERCA CNDSSLDYNEAKGVYEKVGEATETALTCLVEKMNVFDTDLKGLSRIERANACNSVIKQLM
White-tailed_tropicbird_SERCA CNDSSLDYNEAKGVYEKVGEATETALTCLVEKMNVFDTDLKGLSRIERANACNSVIKQLM
Ruff_SERCA CNDSSLDYNEAKGVYEKVGEATETALTCLVEKMNVFDTDLKGLSRIERANACNSVIKQLM
Turkey_SERCA CNDSSLDYNEAKGVYEKVGEATETALTCLVEKMNVFDTDLKGLSRIERANACNSVIKQLM
Little-egret_SERCA CNDSSLDYNEAKGVYEKVGEATETALTCLVEKMNVFDTDLKGLSRIERANACNSVIKQLM
Cuckoo_SERCA CNDSSLDYNEAKGVYEKVGEATETALTCLVEKMNVFDTDLKGLSRIERANACNSVIKQLM
Crowned-crane_SERCA CNDSSLDYNEAKGVYEKVGEATETALTCLVEKMNVFDTDLKGLSRIERANACNSVIKQLM
Chicken_SERCA1 CNDSSLDYNEAKGIYEKVGEATETALTCLVEKMNVFNTDVRSLSKVERANACNSVIKQLM
Sifaka_SERCA1 CNDSSLDFNEAKGVYEKVGEATETALTTLVEKMNVFNTDVKSLSKVERANACNSVIRQLM
Pongo_SERCA1 CNDSSLDFNEAKGVYEKVGEATETALTTLVEKMNVFNTDVRSLSKVERANACNSVIRQLM
Human_SERCA1 CNDSSLDFNEAKGVYEKVGEATETALTTLVEKMNVFNTDVRSLSKVERANACNSVIRQLM
Chimpanzee_SERCA1 CNDSSLDFNEAKGVYEKVGEATETALTTLVEKMNVFNTDVRSLSKVERANACNSVIRQLM
Domestic_ferret_SERCA1 CNDSALDFNETKGVYEKVGEATETALTTLVEKMNVFNTDVRSLSKVERANACNSVIRQLM
Dog_SERCA1 CNDSALDFNETKGVYEKVGEATETALTTLVEKMNVFNTDVRNLSKVERANACNSVIRQLM
Panthera_SERCA1 CNDSSLDFNEAKGVYEKVGEATETALTTLVEKMNVFSTDVRSLSKVERANACNSVIRQLM
Cat_SERCA1 CNDSSLDFNEAKGVYEKVGEATETALTTLVEKMNVFSTDVRSLSKVERANACNSVIRQLM
Horseshoe-bat_SERCA1 CNDSSLDFNEAKGVYEKVGEATETALTTLVEKMNVFNTDVRSLSKVERANACNSVIRQLM
Vampire_bat_SERCA1 CNDSSLDFNEAKGVYEKVGEATETALTTLVEKMNVFNTDVRNLSKVERANACNSVIRQLM
Egyptian_rousette_SERCA1 CNDSSLDFNEAKGVYEKVGEATETALTTLVEKMNVFNTEVRSLSKVERANACNSVIRQLM
Philippine_tarsier_SERCA1 CNDSSLDFNETKGVYEKVGEATETALTTLVEKMNVFNTDVRNLSKVERANACNSVIRQLM
Rabbit_SERCA1 CNDSSLDFNETKGVYEKVGEATETALTTLVEKMNVFNTEVRNLSKVERANACNSVIRQLM
American_pika_SERCA1 CNDSSLDFNESKGVYEKVGEATETALTTLVEKMNVFNTDVRSLSKVERANACNSVIRQLM
Thirteen-lined_ground_squirrel_SERCA1 CNDSSLDFNETKGVYEKVGEATETALTTLVEKMNVFNTDVRSLSKVERANACNSVIRQLM
Alpine_marmot_SERCA1 CNDSSLDFNETKGVYEKVGEATETALTTLVEKMNVFNTDVRSLSKVERANACNSVIRQLM
Chinchilla_SERCA1 CNDSSLDFNEAKGVYEKVGEATETALTTLVEKMNVFNTEVRSLSKVERANACNSVIRQLM
Cape_elephant-shrew_SERCA1 CNDSSLDFNEAKGVYEKVGEATETALTTLVEKMNVFNTDVRSLSKVERANACNSVIRQLM
Chinese_ CNDSSLDFNETKGVYEKVGEATETALTTLVEKMNVFNTDVRSLSKVERANACNSVIRQLM
Kangaroo_rat_SERCA1 CNDSSLDFNETKGVYEKVGEATETALTTLVEKMNVFNTEVRSLSKVERANACNSVIRQLM
Egyptian_jerboa_SERCA1 CNDSSLDFNESKGVYEKVGEATETALTTLVEKMNVFNTEVRSLSKVERANACNSVIRQLM
Mongolian_gerbil_SERCA1 CNDSSLDFNETKGVYEKVGEATETALTTLVEKMNVFNTEVRNLSKVERANACNSVIRQLM
Prairie_vole_SERCA1 CNDSSLDFNETKGVYEKVGEATETALTTLVEKMNVFNTEVRSLSKVERANACNSVIRQLM
Chinese CNDSSLDFNETKGVYEKVGEATETALTTLVEKMNVFNTEVRSLSKVERANACNSVIRQLM
Rattus_SERCA1 CNDSSLDFNETKGVYEKVGEATETALTTLVEKMNVFNTEVRSLSKVERANACNSVIRQLM
Mus_SERCA1 CNDSSLDFNETKGVYEKVGEATETALTTLVEKMNVFNTEVRSLSKVERANACNSVIRQLM

```
                                               480       490       500       510       520       530
Tinamou_SERCA                                  KKEFTLEFSRDRKSMSVYCTPNKPSRTSM.SKMFVKGAPEGVIDRCTHVRVGNAKIPLTS
Hummingbird_SERCA                              KKEFTLEFSRDRKSMSVYCTPNKPSRASM.TKMFVKGAPEGVIDRCTHVRVGNAKIPLTP
Manakin_SERCA                                  KKEFTLEFSRDRKSMSVYCTPNKPSRTSM.SKMFVKGAPEGVIDRCTHVRVGNAKIPLTP
Finch_SERCA1                                   KKEFTLEFSRDRKSMSVYCTPNKPSRTSM.SKMFVKGAPEGVIDRCTHVRVGNAKIPLTP
Sparrow_SERCA                                  KKEFTLEFSRDRKSMSVYCTPNKPSRTSM.SKMFVKGAPEGVIDRCTHVRVGNAKIPLTS
Starling_SERCA                                 KKEFTLEFSRDRKSMSVYCTPNKPSRTSM.SKMFVKGAPEGVIDRCTHVRVGNAKIPLTS
Tibetan_ground-tit_SERCA                       KKEFTLEFSRDRKSMSVYCTPNKPSRTSM.SKMFVKGAPEGVIDRCTHVRVGNAKIPLTS
Ground-finch_SERCA                             KKEFTLEFSRDRKSMSVYCTPNKPSRTSM.SKMFVKGAPEGVIDRCTHVRVGNAKIPLTS
Downy_woodpecker_SERCA                         KKEFTLEFSRDRKSMSVYCTPNKPSRTSM.SKMFVKGAPEGVIDRCTHVRVGSAKIPLTP
Struthio_camelus_SERCA                         KKEFTLEFSRDRKSMSVYCTPNKPSRTSM.SKMFVKGAPEGVIDRCTHVRVGNAKIPLTS
Penguin_SERCA                                  KKEFTLEFSRDRKSMSVYCTPNKPSRTSM.SKMFVKGAPEGVIDRCTHVRAGNAKIPLTS
Adelie_penguin_SERCA                           KKEFTLEFSRDRKSMSVYCTPNKPSRTSM.SKMFVKGAPEGVIDRCTHVRVGNAKIPLTS
Ruber_ruber_SERCA                              KKEFTLEFSRDRKSMSVYCTPNKPSRTSM.SKMFVKGAPEGVIDRCTHVRVGNAKIPLTS
Swift_SERCA                                    KKEFTLEFSRDRKSMSVYCTPNKPSRTSM.SKMFVKGAPEGVIDRCTHVRVGNAKIPLTS
Falcon_SERCA                                   KKEFTLEFSRDRKSMSVYCTPNKPSRTSM.SKMFVKGAPEGVIDRCTHVRVGSAKIQLTS
Japanese-quail_SERCA                           KKEFTLEFSRDRKSMSVYCTLNKPSRTSM.SKMFVKGAPEGVIDRCTHVRVGNAKIPLTS
White-tailed_tropicbird_SERCA                  KKEFTLEFSRDRKSMSVYCTPNKPSRTSM.SKMFVKGAPEGVIDRCTHVRVGNAKILLTA
Ruff_SERCA                                     KKEFTLEFSRDRKSMSVYCTPNKPSRTSM.SKMFVKGAPEGVIDRCTHVRVGNTKIPLTT
Turkey_SERCA                                   KKEFTLEFSRDRKSMSVYCTPNKPSRTSM.SKMFVKGAPEGVIDRCTHVRVGNAKIPLSS
Little-egret_SERCA                             KKEFTLEFSRDRKSMSVYCTPNKPSRTSM.SKMFVKGAPEGVIDRCTHVRVGSAKIPLTS
Cuckoo_SERCA                                   KKEFTLEFSRDRKSMSVYCTPNKPSRTSM.SKMFVKGAPEGVIDRCTHVRVGNAKIPLTS
Crowned-crane_SERCA                            KKEFTLEFSRDRKSMSVYCTPNKPSRTSM.SKMFVKGAPEGVIDRCTHVRVGNAKIPLTS
Chicken_SERCA1                                 KKEFTLEFSRDRKSMSVYCSPAKASRAAVGNKMFVKGAPEGVIDRCNYVRVGTTRVPLTP
Sifaka_SERCA1                                  KKEFTLEFSRDRKSMSVYCSPAKSSRAAVGNKMFVKGAPEGVIDRCNYVRVGTTRVPLTT
Pongo_SERCA1                                   KKEFTLEFSRDRKSMSVYCSPAKSSRAAVGNKMFVKGAPEGVIDRCNYVRVGTTRVPLTG
Human_SERCA1                                   KKEFTLEFSRDRKSMSVYCSPAKSSRAAVGNKMFVKGAPEGVIDRCNYVRVGTTRVPLTG
Chimpanzee_SERCA1                              KKEFTLEFSRDRKSMSVYCSPAKSSRAAVGNKMFVKGAPEGVIDRCNYVRVGTTRVPLTG
Domestic_ferret_SERCA1                         KKEFTLEFSRDRKSMSVYCSPAKS.RAAVGNKMFVKGAPEGVIDRCNYVRVGTTRVPMTG
Dog_SERCA1                                     KKEFTLEFSRDRKSMSVYCSPAKS.RAAVGNKMFVKGAPEGVIDRCNYVRVGTTRVPMTG
Panthera_SERCA1                                KKEFTLEFSRDRKSMSVYCSPAKS.RAAVGNKMFVKGAPEGVIDRCNYVRVGTTRVPITG
Cat_SERCA1                                     KKEFTLEFSRDRKSMSVYCSPAKS.RAAVGNKMFVKGAPEGVIDRCNYVRVGTTRVPITG
Horseshoe-bat_SERCA1                           KKECTLEFSRDRKSMSVYCSPAKS.RAAVGNKMFVKGAPEGVIDRCNYVRVGTTRVPMTG
Vampire_bat_SERCA1                             KKEFTLEFSRDRKSMSVYCSPAKS.RAAVGNKMFVKGAPEGVIDRCNYVRVGTTRVPMTG
Egyptian_rousette_SERCA1                       KKEFTLEFSRDRKSMSVYCSPAKS.RAAVGNKMFVKGAPEGVIDRCNYVRVGTTRVPLTG
Philippine_tarsier_SERCA1                      KKEFTLEFSRDRKSMSVYCSPAKSSRAAVGNKMFIKGAPEGVIDRCNYVRVGTTRVPLTG
Rabbit_SERCA1                                  KKEFTLEFSRDRKSMSVYCSPAKSSRAAVGNKMFVKGAPEGVIDRCNYVRVGTTRVPMTG
American_pika_SERCA1                           KKEFTLEFSRDRKSMSVYCSPAKSSRAAVGNKMFVKGAPEGVIDRCNYVRVGTTRVPMTG
Thirteen-lined_ground_squirrel_SERCA1          KKEFTLEFSRDRKSMSVYCSPAKSSRAAVGNKMFVKGAPEGVIDRCNYVRVGTTRVPLTG
Alpine_marmot_SERCA1                           KKEFTLEFSRDRKSMSVYCSPAKSSRAAVGNKMFVKGAPEGVIDRCNYVRVGTTRVPLTG
Chinchilla_SERCA1                              KKEFTLEFSRDRKSMSVYCSPAKSSRAAVGNKMFVKGAPEGVIDRCNYVRVGTTRVPLTG
Cape_elephant-shrew_SERCA1                     KKEFTLEFSRDRKSMSVYCSPAKSSRAAVGNKMFVKGAPEGVIDRCNYIRVGTTRVPMTG
Chinese_                                       KKEFTLEFSRDRKSMSVYCSPAKSSRAAVGNKMFVKGAPEGVIDRCNYVRVGTTRVPLTG
Kangaroo_rat_SERCA1                            KKEFTLEFSRDRKSMSVYCSPAKSSRAAVGNKMFVKGAPEGVIDRCNYVRVGTTRVPLTG
Egyptian_jerboa_SERCA1                         KKEFTLEFSRDRKSMSVYCSPAKSSRAAVGNKMFVKGAPEGVIDRCNYVRVGTTRVPLTG
Mongolian_gerbil_SERCA1                        KKEFTLEFSRDRKSMSVYCSPAKSSRAAVGNKMFVKGAPEGVIDRCNYVRVGTTRVPLTG
Prairie_vole_SERCA1                            KKEFTLEFSRDRKSMSVYCSPAKSSRAAVGNKMFVKGAPEGVIDRCNYVRVGTTRVPLTG
Chinese                                        KKEFTLEFSRDRKSMSVYCSPAKSSRAAVGNKMFVKGAPEGVIDRCNYVRVGTTRVPLTG
Rattus_SERCA1                                  KKEFTLEFSRDRKSMSVYCSPAKSSRAAVGNKMFVKGAPEGVIDRCNYVRVGTTRVPLTG
Mus_SERCA1                                     KKEFTLEFSRDRKSMSVYCSPAKSSRAAVGNKMFVKGAPEGVIDRCNYVRVGTTRVPLTG
```

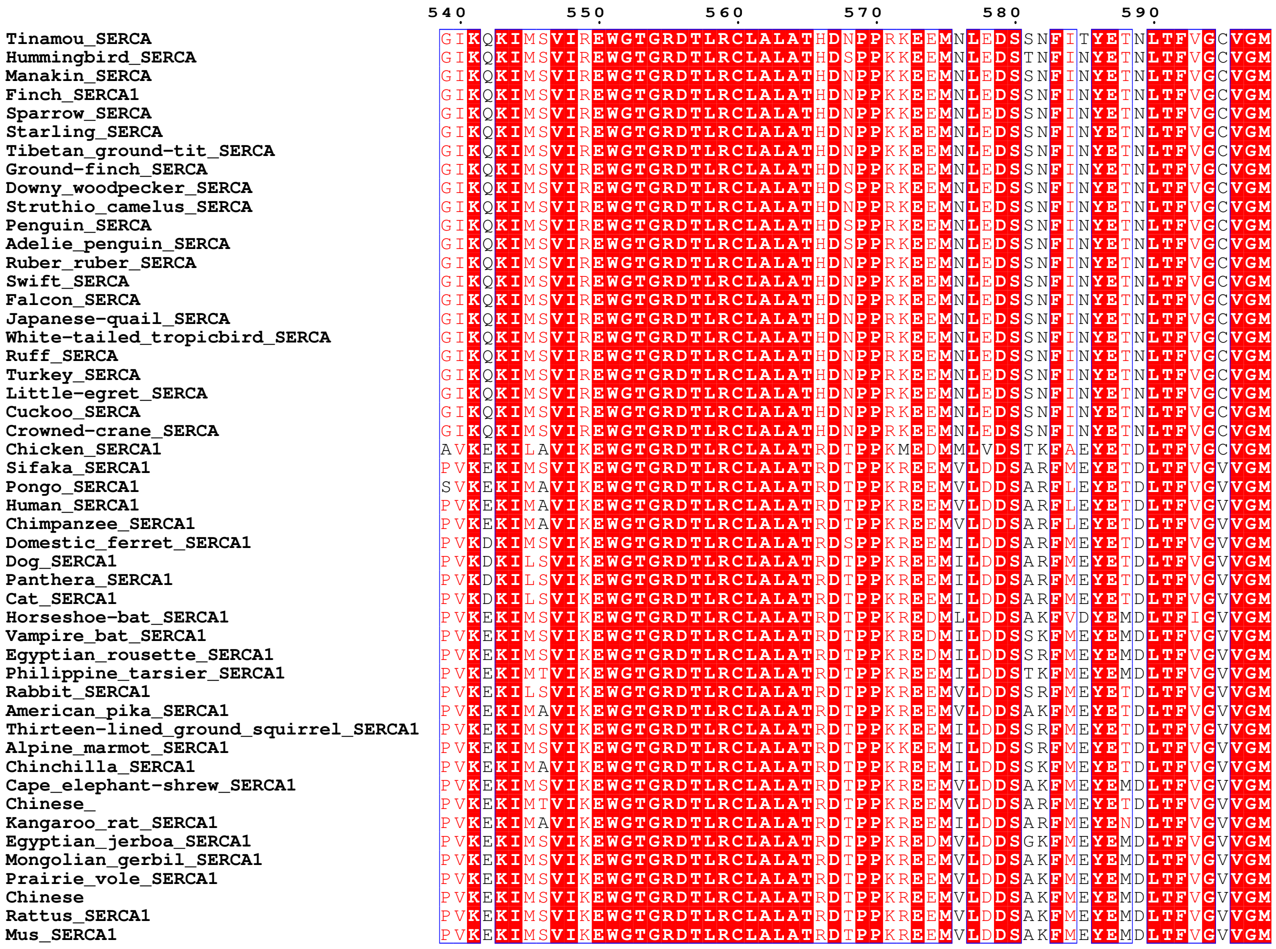
540 550 560 570 580 590
Tinamou_SERCA GIKQKIMSVIREWGTGRDTLRCLALATHDNPPRKEEMNLEDSSNFITYETNLTFVGCVGM
Hummingbird_SERCA GIKQKIMSVIREWGTGRDTLRCLALATHDSPPKKEEMNLEDSTNFINYETNLTFVGCVGM
Manakin_SERCA GIKQKIMSVIREWGTGRDTLRCLALATHDNPPKKEEMNLEDSSNFINYETNLTFVGCVGM
Finch_SERCA1 GIKQKIMSVIREWGTGRDTLRCLALATHDNPPKKEEMNLEDSSNFINYETNLTFVGCVGM
Sparrow_SERCA GIKQKIMSVIREWGTGRDTLRCLALATHDNPPKKEEMNLEDSSNFINYETNLTFVGCVGM
Starling_SERCA GIKQKIMSVIREWGTGRDTLRCLALATHDNPPKKEEMNLEDSSNFINYETNLTFVGCVGM
Tibetan_ground-tit_SERCA GIKQKIMSVIREWGTGRDTLRCLALATHDNPPKKEEMNLEDSSNFINYETNLTFVGCVGM
Ground-finch_SERCA GIKQKIMSVIREWGTGRDTLRCLALATHDNPPKKEEMNLEDSSNFINYETNLTFVGCVGM
Downy_woodpecker_SERCA GIKQKIMSVIREWGTGRDTLRCLALATHDSPPRKEEMNLEDSSNFINYETNLTFVGCVGM
Struthio_camelus_SERCA GIKQKIMSVIREWGTGRDTLRCLALATHDNPPRKEEMNLEDSSNFINYETNLTFVGCVGM
Penguin_SERCA GIKQKIMSVIREWGTGRDTLRCLALATHDSPPRKEEMNLEDSSNFINYETNLTFVGCVGM
Adelie_penguin_SERCA GIKQKIMSVIREWGTGRDTLRCLALATHDSPPRKEEMNLEDSSNFINYETNLTFVGCVGM
Ruber_ruber_SERCA GIKQKIMSVIREWGTGRDTLRCLALATHDNPPRKEEMNLEDSSNFINYETNLTFVGCVGM
Swift_SERCA GIKQKIMSVIREWGTGRDTLRCLALATHDNPPKKEEMNLEDSSNFINYETNLTFVGCVGM
Falcon_SERCA GIKQKIMSVIREWGTGRDTLRCLALATHDNPPRKEEMNLEDSSNFINYETNLTFVGCVGM
Japanese-quail_SERCA GIKQKIMSVIREWGTGRDTLRCLALATHDNPPRKEEMNLEDSSNFINYETNLTFVGCVGM
White-tailed_tropicbird_SERCA GIKQKIMSVIREWGTGRDTLRCLALATHDNPPRKEEMNLEDSSNFINYETNLTFVGCVGM
Ruff_SERCA GIKQKIMSVIREWGTGRDTLRCLALATHDNPPRKEEMNLEDSSNFINYETNLTFVGCVGM
Turkey_SERCA GIKQKIMSVIREWGTGRDTLRCLALATHDNPPRKEEMNLEDSSNFINYETNLTFVGCVGM
Little-egret_SERCA GIKQKIMSVIREWGTGRDTLRCLALATHDNPPRKEEMNLEDSSNFINYETNLTFVGCVGM
Cuckoo_SERCA GIKQKIMSVIREWGTGRDTLRCLALATHDNPPRKEEMNLEDSSNFINYETNLTFVGCVGM
Crowned-crane_SERCA GIKQKIMSVIREWGTGRDTLRCLALATHDNPPRKEEMNLEDSSNFINYETNLTFVGCVGM
Chicken_SERCA1 AVKEKILAVIKEWGTGRDTLRCLALATRDTPPKMEDMMLVDSTKFAEYETDLTFVGCVGM
Sifaka_SERCA1 PVKEKIMSVIKEWGTGRDTLRCLALATRDTPPKREEMVLDDSARFMEYETDLTFVGVVGM
Pongo_SERCA1 SVKEKIMAVIKEWGTGRDTLRCLALATRDTPPKREEMVLDDSARFLEYETDLTFVGVVGM
Human_SERCA1 PVKEKIMAVIKEWGTGRDTLRCLALATRDTPPKREEMVLDDSARFLEYETDLTFVGVVGM
Chimpanzee_SERCA1 PVKEKIMAVIKEWGTGRDTLRCLALATRDTPPKREEMVLDDSARFLEYETDLTFVGVVGM
Domestic_ferret_SERCA1 PVKDKIMSVIKEWGTGRDTLRCLALATRDSPPKREEMILDDSARFMEYETDLTFVGVVGM
Dog_SERCA1 PVKDKILSVIKEWGTGRDTLRCLALATRDTPPKREEMILDDSARFMEYETDLTFVGVVGM
Panthera_SERCA1 PVKDKILSVIKEWGTGRDTLRCLALATRDTPPKREEMILDDSARFMEYETDLTFVGVVGM
Cat_SERCA1 PVKDKILSVIKEWGTGRDTLRCLALATRDTPPKREEMILDDSARFMEYETDLTFVGVVGM
Horseshoe-bat_SERCA1 PVKEKIMSVIKEWGTGRDTLRCLALATRDTPPKREDMLLDDSAKFVDYEMDLTFIGVVGM
Vampire_bat_SERCA1 PVKEKIMSVIKEWGTGRDTLRCLALATRDTPPKREDMILDDSSKFMEYEMDLTFVGVVGM
Egyptian_rousette_SERCA1 PVKEKIMSVIKEWGTGRDTLRCLALATRDTPPKREDMILDDSSRFMEYEMDLTFVGVVGM
Philippine_tarsier_SERCA1 PVKEKIMTVIKEWGTGRDTLRCLALATRDTPPKREEMILDDSTKFMEYEMDLTFVGVVGM
Rabbit_SERCA1 PVKEKILSVIKEWGTGRDTLRCLALATRDTPPKREEMVLDDSSRFMEYETDLTFVGVVGM
American_pika_SERCA1 PVKEKIMAVIKEWGTGRDTLRCLALATRDTPPKREEMVLDDSAKFMEYETDLTFVGVVGM
Thirteen-lined_ground_squirrel_SERCA1 PVKEKIMSVIKEWGTGRDTLRCLALATRDTPPKKEEMILDDSSRFMEYETDLTFVGVVGM
Alpine_marmot_SERCA1 PVKEKIMSVIKEWGTGRDTLRCLALATRDTPPKKEEMILDDSSRFMEYETDLTFVGVVGM
Chinchilla_SERCA1 PVKEKIMAVIKEWGTGRDTLRCLALATRDTPPKREEMILDDSSKFMEYETDLTFVGVVGM
Cape_elephant-shrew_SERCA1 PVKEKIMSVIKEWGTGRDTLRCLALATRDTPPKREEMVLDDSAKFMEYEMDLTFVGVVGM
Chinese_ PVKEKIMTVIKEWGTGRDTLRCLALATRDTPPKREEMVLDDSARFMEYETDLTFVGVVGM
Kangaroo_rat_SERCA1 PVKEKIMAVIKEWGTGRDTLRCLALATRDTPPKREEMILDDSARFMEYENDLTFVGVVGM
Egyptian_jerboa_SERCA1 PVKEKIMSVIKEWGTGRDTLRCLALATRDTPPKREDMVLDDSGKFMEYEMDLTFVGVVGM
Mongolian_gerbil_SERCA1 PVKEKIMSVIKEWGTGRDTLRCLALATRDTPPKREEMVLDDSAKFMEYEMDLTFVGVVGM
Prairie_vole_SERCA1 PVKEKIMSVIKEWGTGRDTLRCLALATRDTPPKREEMVLDDSAKFMEYEMDLTFVGVVGM
Chinese PVKEKIMSVIKEWGTGRDTLRCLALATRDTPPKREEMVLDDSAKFMEYEMDLTFVGVVGM
Rattus_SERCA1 PVKEKIMSVIKEWGTGRDTLRCLALATRDTPPKREEMVLDDSAKFMEYEMDLTFVGVVGM
Mus_SERCA1 PVKEKIMSVIKEWGTGRDTLRCLALATRDTPPKREEMVLDDSAKFMEYEMDLTFVGVVGM

```
                                    600       610       620       630       640       650
                                      .         .         .         .         .         .
Tinamou_SERCA                       LDPPRIEVASSIKLCKQAGIRVIMITGDNKGTAVAICRRIGIFVEDEDVSTKAFTGREFD
Hummingbird_SERCA                   LDPPRIEVASSIKLCRQAGIRVIMITGDNKGTAVAICRRIGIFVEDEDVSTKAFTGREFD
Manakin_SERCA                       LDPPRIEVASSIKLCRQAGIRVIMITGDNKGTAVAICRRIGIFVEDEDVSTKAFTGREFD
Finch_SERCA1                        LDPPRIEVASSIKLCRQAGIRVIMITGDNKGTAVAICRRIGIFVEDEDVSTKAFTGREFD
Sparrow_SERCA                       LDPPRIEVASSIKLCRQAGIRVIMITGDNKGTAVAICRRIGIFVEDEDVSSKAFTGREFD
Starling_SERCA                      LDPPRIEVASSIKLCRQAGIRVIMITGDNKGTAVAICRRIGIFVEDEDVSTKAFTGREFD
Tibetan_ground-tit_SERCA            LDPPRIEVASSIKLCRQAGIRVIMITGDNKGTAVAICRRIGIFVEDEDVSTKAFTGREFD
Ground-finch_SERCA                  LDPPRIEVASSIKLCRQAGIRVIMITGDNKGTAVAICRRIGIFVEDEDVSTKAFTGREFD
Downy_woodpecker_SERCA              LDPPRIEVASSIKLCRQAGIRVIMITGDNKGTAVAICRRIGIFVEDEDVSTKAFTGREFD
Struthio_camelus_SERCA              LDPPRIEVASSIKLCKQAGIRVIMITGDNKGTAVAICRRIGIFVEDEDVSTKAFTGREFD
Penguin_SERCA                       LDPPRIEVASSIKLCKQAGIRVIMITGDNKGTAVAICRRIGIFVEDEDVSTKAFTGREFD
Adelie_penguin_SERCA                LDPPRIEVASSIKLCKQAGIRVIMITGDNKGTAVAICRRIGIFVEDEDVSTKAFTGREFD
Ruber_ruber_SERCA                   LDPPRSEVASSIKLCKQAGIRVIMITGDNKGTAVAICRRIGIFVEDEDVSTKAFTGREFD
Swift_SERCA                         LDPPRIEVASSIKLCKQAGIRVIMITGDNKGTAVAICRRIGIFVEDEDVSTKAFTGREFD
Falcon_SERCA                        LDPPRIEVASSIKLCKQAGIRVIMITGDNKGTAVAICRRIGIFVEDEDVSTKAFTGREFD
Japanese-quail_SERCA                LDPPRIEVASSIKLCKQAGIRVIMITGDNKGTAVAICRRIGIFVEDEDVSTKAFTGREFD
White-tailed_tropicbird_SERCA       LDPPRIEVASSIKLCKQAGIRVIMITGDNKGTAVAICRRIGIFVEDEDVSTKAFTGREFD
Ruff_SERCA                          LDPPRIEVASSIKLCKQAGIRVIMITGDNKGTAVAICRRIGIFVEDEDVSTKAFTGREFD
Turkey_SERCA                        LDPPRIEVASSIKLCKQAGIRVIMITGDNKGTAVAICRRIGIFVEDEDVSTKAFTGREFD
Little-egret_SERCA                  LDPPRIEVASSIKLCKQAGIRVIMITGDNKGTAVAICRRIGIFVEDEDVSTKAFTGREFD
Cuckoo_SERCA                        LDPPRIEVASSIKLCKQAGIRVIMITGDNKGTAVAICRRIGIFVEDEDVSTKAFTGREFD
Crowned-crane_SERCA                 LDPPRIEVASSIKLCKQAGIRVIMITGDNKGTAVAICRRIGIFVEDEDVSTKAFTGREFD
Chicken_SERCA1                      LDPPRKEVMGSIRLCRDAGIRVIMITGDNKGTAIAICRRIGIFTEDEEVSGRAYTGREFD
Sifaka_SERCA1                       LDPPRKEVTGSIQLCRDAGIRVIMITGDNKGTAIAICRRIGIFGENEEVADRAYTGREFD
Pongo_SERCA1                        LDPPRKEVTGSIQLCRDAGIRVIMITGDNKGTAIAICQRIGIFGENEEVADRAYTGREFD
Human_SERCA1                        LDPPRKEVTGSIQLCRDAGIRVIMITGDNKGTAIAICRRIGIFGENEEVADRAYTGREFD
Chimpanzee_SERCA1                   LDPPRKEVTGSIQLCRDAGIRVIMITGDNKGTAIAICRRIGIFGENEEVADRAYTGREFD
Domestic_ferret_SERCA1              LDPPRKEVTGSIQLCRDAGIRVIMITGDNKGTAIAICRRIGIFGENEEVTDRAYTGREFD
Dog_SERCA1                          LDPPRKEVTGSIQLCRDAGIRVIMITGDNKGTAIAICRRIGIFGENEEVADRAYTGREFD
Panthera_SERCA1                     LDPPRKEVTGSIQLCRDAGIRVIMITGDNKGTAIAICRRIGIFGENEEVADRAYTGREFD
Cat_SERCA1                          LDPPRKEVTGSIQLCRDAGIRVIMITGDNKGTAIAICRRIGIFGENEEVADRAYTGREFD
Horseshoe-bat_SERCA1                LDPPRKEVTGSIQLCRDAGIRVIMITGDNKGTAIAICRRIGIFGENEDVADRAYTGREFD
Vampire_bat_SERCA1                  LDPPRKEVTGSIQLCRDAGIRVIMITGDNKGTAIAICRRIGIFGENEEVADRAYTGREFD
Egyptian_rousette_SERCA1            LDPPRKEVTGSIQLCRDAGIRVIMITGDNKGTAIAICRRIGIFGENEEVADRAYTGREFD
Philippine_tarsier_SERCA1           LDPPRKEVTGSIQLCRDAGIRVIMITGDNKGTAIAICRRIGIFGENEEVTDRAYTGREFD
Rabbit_SERCA1                       LDPPRKEVMGSIQLCRDAGIRVIMITGDNKGTAIAICRRIGIFGENEEVADRAYTGREFD
American_pika_SERCA1                LDPPRKEVMGSIQLCRDAGIRVIMITGDNKGTAIAICRRIGIFGENEEVTDRAYTGREFD
Thirteen-lined_ground_squirrel_SERCA1 LDPPRKEVTGSIQLCRDAGIRVIMITGDNKGTAIAICRRIGIFSESEQVTDRAYTGREFD
Alpine_marmot_SERCA1                LDPPRKEVTGSIQLCRDAGIRVIMITGDNKGTAIAICRRIGIFSESEQVTDRAYTGREFD
Chinchilla_SERCA1                   LDPPRKEVTGSIQLCRDAGIRVIMITGDNKGTAIAICRRIGIFSESEEVADRAYTGREFD
Cape_elephant-shrew_SERCA1          LDPPRKEVTGSIQLCRDAGIRVIMITGDNKGTAIAICRRIGIFSENEEVADRAYTGREFD
Chinese_                            LDPPRKEVTGSIQLCRDAGIRVIMITGDNKGTAIAICRRIGIFGENEEVADRAYTGREFD
Kangaroo_rat_SERCA1                 LDPPRKEVTGSIQLCRDAGIRVIMITGDNKGTAIAICRRIGIFSESEEVTDRAYTGREFD
Egyptian_jerboa_SERCA1              LDPPRKEVTGSIQLCRDAGIRVIMITGDNKGTAIAICRRIGIFSENEEVTDRAYTGREFD
Mongolian_gerbil_SERCA1             LDPPRKEVTGSIQLCRDAGIRVIMITGDNKGTAIAICRRIGIFSENEEVADRAYTGREFD
Prairie_vole_SERCA1                 LDPPRKEVTGSIQLCRDAGIRVIMITGDNKGTAIAICRRIGIFSENEEVTDRAYTGREFD
Chinese                             LDPPRKEVTGSIQLCRDAGIRVIMITGDNKGTAIAICRRIGIFSENEEVTDRAYTGREFD
Rattus_SERCA1                       LDPPRKEVTGSIQLCRDAGIRVIMITGDNKGTAIAICRRIGIFSENEEVADRAYTGREFD
Mus_SERCA1                          LDPPRKEVTGSIQLCRDAGIRVIMITGDNKGTAIAICRRIGIFSENEEVTDRAYTGREFD
```

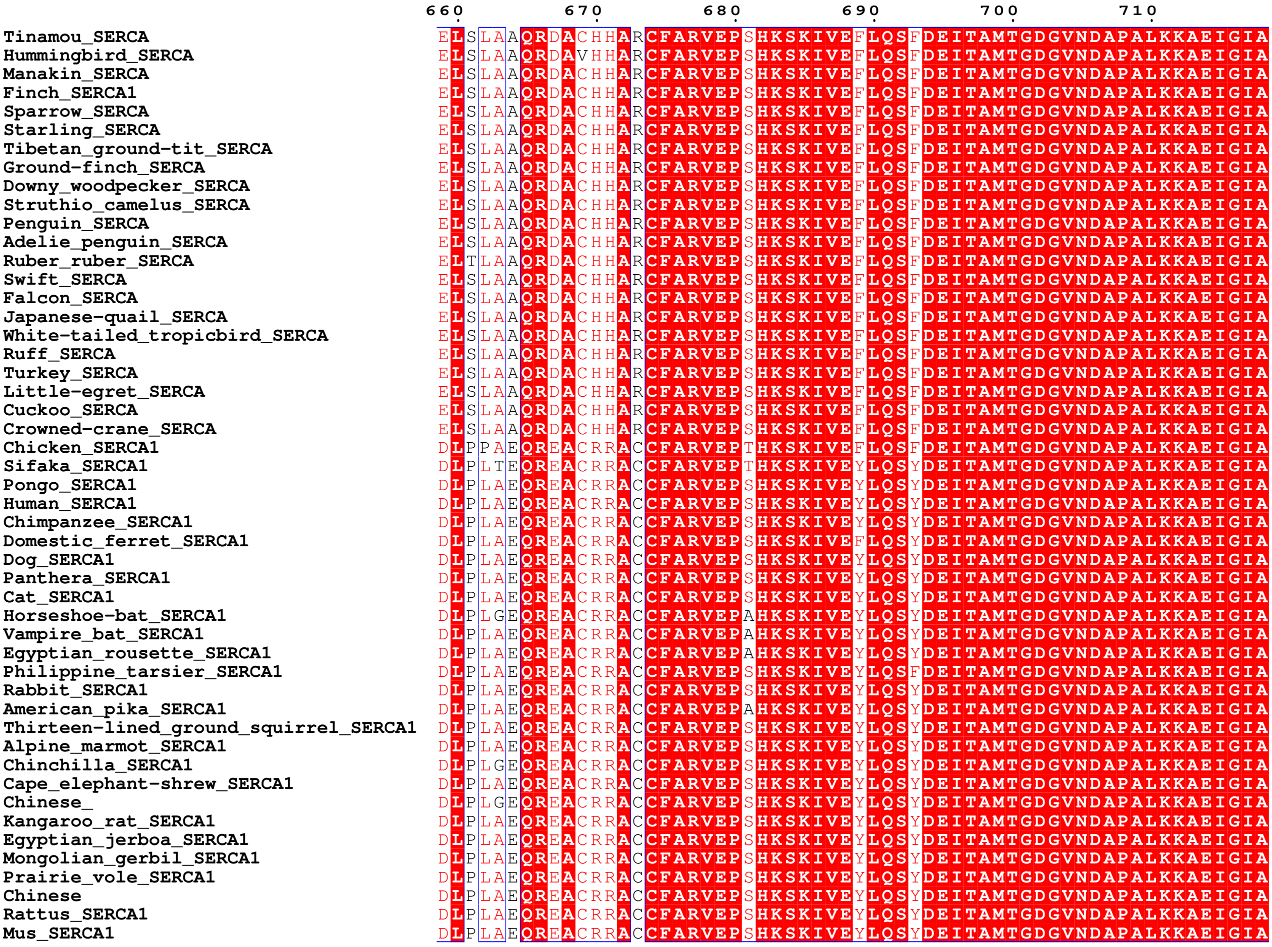
660 670 680 690 700 710
Tinamou_SERCA ELSLAAQRDACHHARCFARVEPSHKSKIVEFLQSFDEITAMTGDGVNDAPALKKAEIGIA
Hummingbird_SERCA ELSLAAQRDAVHHARCFARVEPSHKSKIVEFLQSFDEITAMTGDGVNDAPALKKAEIGIA
Manakin_SERCA ELSLAAQRDACHHARCFARVEPSHKSKIVEFLQSFDEITAMTGDGVNDAPALKKAEIGIA
Finch_SERCA1 ELSLAAQRDACHHARCFARVEPSHKSKIVEFLQSFDEITAMTGDGVNDAPALKKAEIGIA
Sparrow_SERCA ELSLAAQRDACHHARCFARVEPSHKSKIVEFLQSFDEITAMTGDGVNDAPALKKAEIGIA
Starling_SERCA ELSLAAQRDACHHARCFARVEPSHKSKIVEFLQSFDEITAMTGDGVNDAPALKKAEIGIA
Tibetan_ground-tit_SERCA ELSLAAQRDACHHARCFARVEPSHKSKIVEFLQSFDEITAMTGDGVNDAPALKKAEIGIA
Ground-finch_SERCA ELSLAAQRDACHHARCFARVEPSHKSKIVEFLQSFDEITAMTGDGVNDAPALKKAEIGIA
Downy_woodpecker_SERCA ELSLAAQRDACHHARCFARVEPSHKSKIVEFLQSFDEITAMTGDGVNDAPALKKAEIGIA
Struthio_camelus_SERCA ELSLAAQRDACHHARCFARVEPSHKSKIVEFLQSFDEITAMTGDGVNDAPALKKAEIGIA
Penguin_SERCA ELSLAAQRDACHHARCFARVEPSHKSKIVEFLQSFDEITAMTGDGVNDAPALKKAEIGIA
Adelie_penguin_SERCA ELSLAAQRDACHHARCFARVEPSHKSKIVEFLQSFDEITAMTGDGVNDAPALKKAEIGIA
Ruber_ruber_SERCA ELTLAAQRDACHHARCFARVEPSHKSKIVEFLQSFDEITAMTGDGVNDAPALKKAEIGIA
Swift_SERCA ELSLAAQRDACHHARCFARVEPSHKSKIVEFLQSFDEITAMTGDGVNDAPALKKAEIGIA
Falcon_SERCA ELSLAAQRDACHHARCFARVEPSHKSKIVEFLQSFDEITAMTGDGVNDAPALKKAEIGIA
Japanese-quail_SERCA ELSLAAQRDACHHARCFARVEPSHKSKIVEFLQSFDEITAMTGDGVNDAPALKKAEIGIA
White-tailed_tropicbird_SERCA ELSLAAQRDACHHARCFARVEPSHKSKIVEFLQSFDEITAMTGDGVNDAPALKKAEIGIA
Ruff_SERCA ELSLAAQRDACHHARCFARVEPSHKSKIVEFLQSFDEITAMTGDGVNDAPALKKAEIGIA
Turkey_SERCA ELSLAAQRDACHHARCFARVEPSHKSKIVEFLQSFDEITAMTGDGVNDAPALKKAEIGIA
Little-egret_SERCA ELSLAAQRDACHHARCFARVEPSHKSKIVEFLQSFDEITAMTGDGVNDAPALKKAEIGIA
Cuckoo_SERCA ELSLAAQRDACHHARCFARVEPSHKSKIVEFLQSFDEITAMTGDGVNDAPALKKAEIGIA
Crowned-crane_SERCA ELSLAAQRDACHHARCFARVEPSHKSKIVEFLQSFDEITAMTGDGVNDAPALKKAEIGIA
Chicken_SERCA1 DLPPAEQREACRRACCFARVEPTHKSKIVEFLQSFDEITAMTGDGVNDAPALKKAEIGIA
Sifaka_SERCA1 DLPLTEQREACRRACCFARVEPTHKSKIVEYLQSYDEITAMTGDGVNDAPALKKAEIGIA
Pongo_SERCA1 DLPLAEQREACRRACCFARVEPSHKSKIVEYLQSYDEITAMTGDGVNDAPALKKAEIGIA
Human_SERCA1 DLPLAEQREACRRACCFARVEPSHKSKIVEYLQSYDEITAMTGDGVNDAPALKKAEIGIA
Chimpanzee_SERCA1 DLPLAEQREACRRACCFARVEPSHKSKIVEYLQSYDEITAMTGDGVNDAPALKKAEIGIA
Domestic_ferret_SERCA1 DLPLAEQREACRRACCFARVEPSHKSKIVEFLQSYDEITAMTGDGVNDAPALKKAEIGIA
Dog_SERCA1 DLPLAEQREACRRACCFARVEPSHKSKIVEYLQSYDEITAMTGDGVNDAPALKKAEIGIA
Panthera_SERCA1 DLPLAEQREACRRACCFARVEPSHKSKIVEYLQSYDEITAMTGDGVNDAPALKKAEIGIA
Cat_SERCA1 DLPLAEQREACRRACCFARVEPSHKSKIVEYLQSYDEITAMTGDGVNDAPALKKAEIGIA
Horseshoe-bat_SERCA1 DLPLGEQREACRRACCFARVEPAHKSKIVEYLQSYDEITAMTGDGVNDAPALKKAEIGIA
Vampire_bat_SERCA1 DLPLAEQREACRRACCFARVEPAHKSKIVEYLQSYDEITAMTGDGVNDAPALKKAEIGIA
Egyptian_rousette_SERCA1 DLPLAEQREACRRACCFARVEPAHKSKIVEYLQSYDEITAMTGDGVNDAPALKKAEIGIA
Philippine_tarsier_SERCA1 DLPLAEQREACRRACCFARVEPSHKSKIVEYLQSFDEITAMTGDGVNDAPALKKAEIGIA
Rabbit_SERCA1 DLPLAEQREACRRACCFARVEPSHKSKIVEYLQSYDEITAMTGDGVNDAPALKKAEIGIA
American_pika_SERCA1 DLPLAEQREACRRACCFARVEPAHKSKIVEYLQSYDEITAMTGDGVNDAPALKKAEIGIA
Thirteen-lined_ground_squirrel_SERCA1 DLPLAEQREACRRACCFARVEPSHKSKIVEYLQSYDEITAMTGDGVNDAPALKKAEIGIA
Alpine_marmot_SERCA1 DLPLAEQREACRRACCFARVEPSHKSKIVEYLQSYDEITAMTGDGVNDAPALKKAEIGIA
Chinchilla_SERCA1 DLPLGEQREACRRACCFARVEPSHKSKIVEYLQSYDEITAMTGDGVNDAPALKKAEIGIA
Cape_elephant-shrew_SERCA1 DLPLAEQREACRRACCFARVEPSHKSKIVEYLQSYDEITAMTGDGVNDAPALKKAEIGIA
Chinese_ DLPLAEQREACRRACCFARVEPSHKSKIVEYLQSYDEITAMTGDGVNDAPALKKAEIGIA
Kangaroo_rat_SERCA1 DLPLAEQREACRRACCFARVEPSHKSKIVEYLQSYDEITAMTGDGVNDAPALKKAEIGIA
Egyptian_jerboa_SERCA1 DLPLAEQREACRRACCFARVEPSHKSKIVEYLQSYDEITAMTGDGVNDAPALKKAEIGIA
Mongolian_gerbil_SERCA1 DLPLAEQREACRRACCFARVEPSHKSKIVEYLQSYDEITAMTGDGVNDAPALKKAEIGIA
Prairie_vole_SERCA1 DLPLAEQREACRRACCFARVEPSHKSKIVEYLQSYDEITAMTGDGVNDAPALKKAEIGIA
Chinese DLPLAEQREACRRACCFARVEPSHKSKIVEYLQSYDEITAMTGDGVNDAPALKKAEIGIA
Rattus_SERCA1 DLPLAEQREACRRACCFARVEPSHKSKIVEYLQSYDEITAMTGDGVNDAPALKKAEIGIA
Mus_SERCA1 DLPLAEQREACRRACCFARVEPSHKSKIVEYLQSYDEITAMTGDGVNDAPALKKAEIGIA

```
                                         720       730       740       750       760       770
Tinamou_SERCA                            MGSGTAVAKTASEMVLADDNFSTIVAAVEEGRAIYNNMKQFIRYLISSNVGEVVCIFLTA
Hummingbird_SERCA                        MGSGTAVAKTASEMVLADDNFSTIVAAVEEGRAIYNNMKQFIRYLISSNVGEVVCIFLTA
Manakin_SERCA                            MGSGTAVAKTASEMVLADDNFSTIVAAVEEGRAIYNNMKQFIRYLISSNVGEVVCIFLTA
Finch_SERCA1                             MGSGTAVAKTASEMVLADDNFSTIVAAVEEGRAIYNNMKQFIRYLISSNVGEVVCIFLTA
Sparrow_SERCA                            MGSGTAVAKTASEMVLADDNFSTIVAAVEEGRAIYNNMKQFIRYLISSNVGEVVCIFLTA
Starling_SERCA                           MGSGTAVAKTASEMVLADDNFSTIVAAVEEGRAIYNNMKQFIRYLISSNVGEVVCIFLTA
Tibetan_ground-tit_SERCA                 MGSGTAVAKTASEMVLADDNFSTIVAAVEEGRAIYNNMKQFIRYLISSNVGEVVCIFLTA
Ground-finch_SERCA                       MGSGTAVAKTASEMVLADDNFSTIVAAVEEGRAIYNNMKQFIRYLISSNVGEVVCIFLTA
Downy_woodpecker_SERCA                   MGSGTAVAKTASEMVLADDNFSTIVAAVEEGRAIYNNMKQFIRYLISSNVGEVVCIFLTA
Struthio_camelus_SERCA                   MGSGTAVAKTASEMVLADDNFSTIVAAVEEGRAIYNNMKQFIRYLISSNVGEVVCIFLTA
Penguin_SERCA                            MGSGTAVAKTASEMVLADDNFSTIVAAVEEGRAIYNNMKQFIRYLISSNVGEVVCIFLTA
Adelie_penguin_SERCA                     MGSGTAVAKTASEMVLADDNFSTIVAAVEEGRAIYNNMKQFIRYLISSNVGEVVCIFLTA
Ruber_ruber_SERCA                        MGSGTAVAKTASEMVLADDNFSTIVAAVEEGRAIYNNMKQFIRYLISSNVGEVVCIFLTA
Swift_SERCA                              MGSGTAVAKTASEMVLADDNFSTIVAAVEEGRAIYNNMKQFIRYLISSNVGEVVCIFLTA
Falcon_SERCA                             MGSGTAVAKTASEMVLADDNFSTIVAAVEEGRAIYNNMKQFIRYLISSNVGEVVCIFLTA
Japanese-quail_SERCA                     MGSGTAVAKTASEMVLADDNFSTIVAAVEEGRAIYNNMKQFIRYLISSNVGEVVCIFLTA
White-tailed_tropicbird_SERCA            MGSGTAVAKTASEMVLADDNFSTIVAAVEEGRAIYNNMKQFIRYLISSNVGEVVCIFLTA
Ruff_SERCA                               MGSGTAVAKTASEMVLADDNFSTIVAAVEEGRAIYNNMKQFIRYLISSNVGEVVCIFLTA
Turkey_SERCA                             MGSGTAVAKTASEMVLADDNFSTIVAAVEEGRAIYNNMKQFIRYLISSNVGEVVCIFLTA
Little-egret_SERCA                       MGSGTAVAKTASEMVLADDNFSTIVAAVEEGRAIYNNMKQFIRYLISSNVGEVVCIFLTA
Cuckoo_SERCA                             MGSGTAVAKTASEMVLADDNFSTIVAAVEEGRAIYNNMKQFIRYLISSNVGEVVCIFLTA
Crowned-crane_SERCA                      MGSGTAVAKTASEMVLADDNFSTIVAAVEEGRAIYNNMKQFIRYLISSNVGEVVCIFLTA
Chicken_SERCA1                           MGSGTAVAKTASEMVLADDNFSTIVAAVEEGRAIYNNMKQFIRYLISSNVGEVVCIFLTA
Sifaka_SERCA1                            MGSGTAVAKTASEMVLADDNFSTIVAAVEEGRAIYNNMKQFIRYLISSNVGEVVCIFLTA
Pongo_SERCA1                             MGSGTAVAKTASEMVLADDNFSTIVAAVEEGRAIYNNMKQFIRYLISSNVGEVVCIFLTA
Human_SERCA1                             MGSGTAVAKTASEMVLADDNFSTIVAAVEEGRAIYNNMKQFIRYLISSNVGEVVCIFLTA
Chimpanzee_SERCA1                        MGSGTAVAKTASEMVLADDNFSTIVAAVEEGRAIYNNMKQFIRYLISSNVGEVVCIFLTA
Domestic_ferret_SERCA1                   MGSGTAVAKTASEMVLADDNFSTIVAAVEEGRAIYNNMKQFIRYLISSNVGEVVCIFLTA
Dog_SERCA1                               MGSGTAVAKTASEMVLADDNFSTIVAAVEEGRAIYNNMKQFIRYLISSNVGEVVCIFLTA
Panthera_SERCA1                          MGSGTAVAKTASEMVLADDNFSTIVAAVEEGRAIYNNMKQFIRYLISSNVGEVVCIFLTA
Cat_SERCA1                               MGSGTAVAKTASEMVLADDNFSTIVAAVEEGRAIYNNMKQFIRYLISSNVGEVVCIFLTA
Horseshoe-bat_SERCA1                     MGSGTAVAKTASEMVLADDNFSTIVAAVEEGRAIYNNMKQFIRYLISSNVGEVVCIFLTA
Vampire_bat_SERCA1                       MGSGTAVAKTASEMVLADDNFSTIVAAVEEGRAIYNNMKQFIRYLISSNVGEVVCIFLTA
Egyptian_rousette_SERCA1                 MGSGTAVAKTASEMVLADDNFSTIVAAVEEGRAIYNNMKQFIRYLISSNVGEVVCIFLTA
Philippine_tarsier_SERCA1                MGSGTAVAKTASEMVLADDNFSTIVAAVEEGRAIYNNMKQFIRYLISSNVGEVVCIFLTA
Rabbit_SERCA1                            MGSGTAVAKTASEMVLADDNFSTIVAAVEEGRAIYNNMKQFIRYLISSNVGEVVCIFLTA
American_pika_SERCA1                     MGSGTAVAKTASEMVLADDNFSTIVAAVEEGRAIYNNMKQFIRYLISSNVGEVVCIFLTA
Thirteen-lined_ground_squirrel_SERCA1    MGSGTAVAKTASEMVLADDNFSTIVAAVEEGRAIYNNMKQFIRYLISSNVGEVVCIFLTA
Alpine_marmot_SERCA1                     MGSGTAVAKTASEMVLADDNFSTIVAAVEEGRAIYNNMKQFIRYLISSNVGEVVCIFLTA
Chinchilla_SERCA1                        MGSGTAVAKTASEMVLADDNFSTIVAAVEEGRAIYNNMKQFIRYLISSNVGEVVCIFLTA
Cape_elephant-shrew_SERCA1               MGSGTAVAKTASEMVLADDNFSTIVAAVEEGRAIYNNMKQFIRYLISSNVGEVVCIFLTA
Chinese_                                 MGSGTAVAKTASEMVLADDNFSTSVAAVEEGRAIYNNMKQFIRYLISSNVGEVVCIFLTA
Kangaroo_rat_SERCA1                      MGSGTAVAKTASEMVLADDNFSTIVAAVEEGRAIYNNMKQFIRYLISSNVGEVVCIFLTA
Egyptian_jerboa_SERCA1                   MGSGTAVAKTASEMVLADDNFSTIVAAVEEGRAIYNNMKQFIRYLISSNVGEVVCIFLTA
Mongolian_gerbil_SERCA1                  MGSGTAVAKTASEMVLADDNFSTIVAAVEEGRAIYNNMKQFIRYLISSNVGEVVCIFLTA
Prairie_vole_SERCA1                      MGSGTAVAKTASEMVLADDNFSTIVAAVEEGRAIYNNMKQFIRYLISSNVGEVVCIFLTA
Chinese                                  MGSGTAVAKTASEMVLADDNFSTIVAAVEEGRAIYNNMKQFIRYLISSNVGEVVCIFLTA
Rattus_SERCA1                            MGSGTAVAKTASEMVLADDNFSTIVAAVEEGRAIYNNMKQFIRYLISSNVGEVVCIFLTA
Mus_SERCA1                               MGSGTAVAKTASEMVLADDNFSTIVAAVEEGRAIYNNMKQFIRYLISSNVGEVVCIFLTA
```

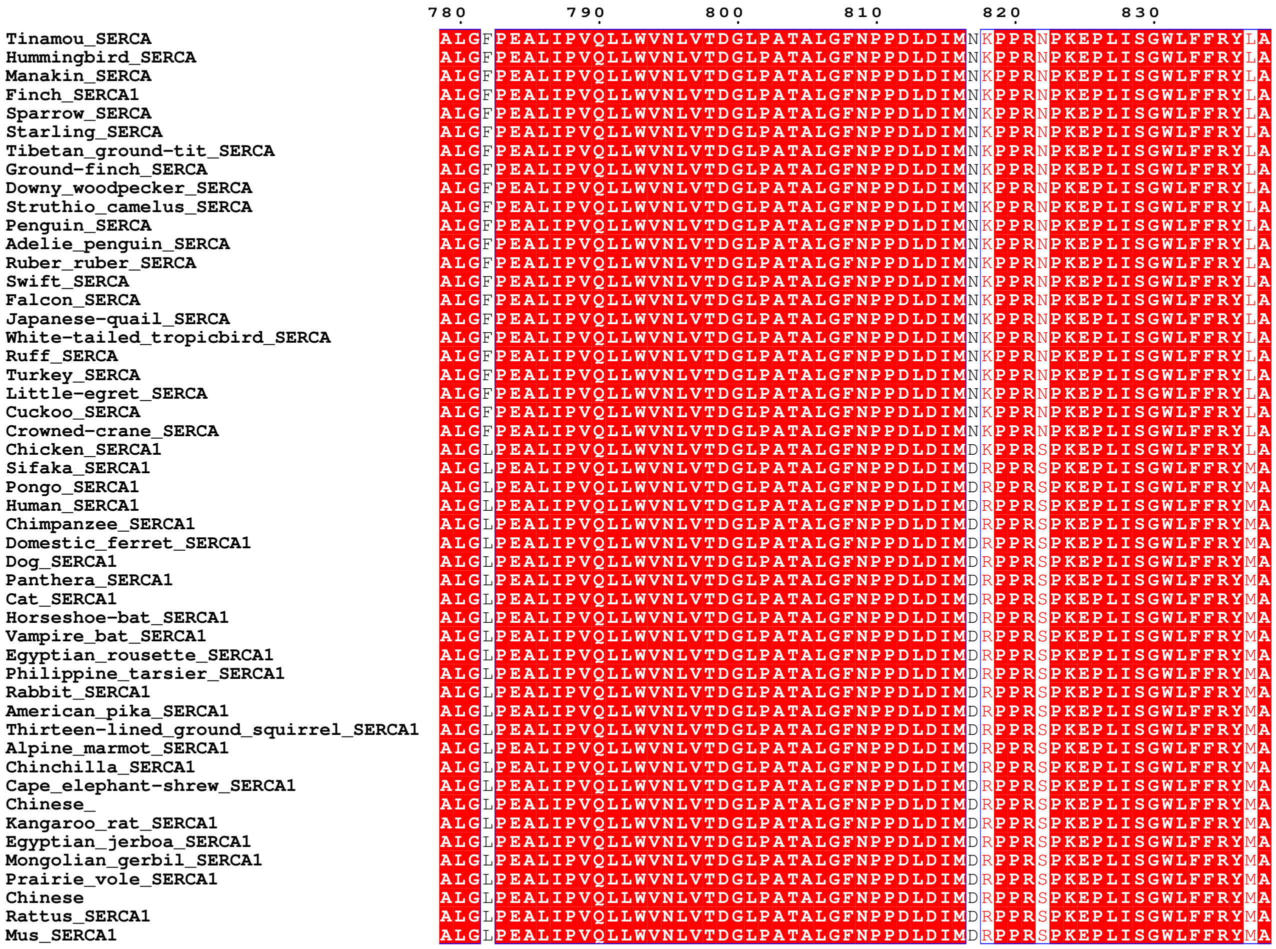
780 790 800 810 820 830
Tinamou_SERCA ALGFPEALIPVQLLWVNLVTDGLPATALGFNPPDLDIMNKPPRNPKEPLISGWLFFRYLA
Hummingbird_SERCA ALGFPEALIPVQLLWVNLVTDGLPATALGFNPPDLDIMNKPPRNPKEPLISGWLFFRYLA
Manakin_SERCA ALGFPEALIPVQLLWVNLVTDGLPATALGFNPPDLDIMNKPPRNPKEPLISGWLFFRYLA
Finch_SERCA1 ALGFPEALIPVQLLWVNLVTDGLPATALGFNPPDLDIMNKPPRNPKEPLISGWLFFRYLA
Sparrow_SERCA ALGFPEALIPVQLLWVNLVTDGLPATALGFNPPDLDIMNKPPRNPKEPLISGWLFFRYLA
Starling_SERCA ALGFPEALIPVQLLWVNLVTDGLPATALGFNPPDLDIMNKPPRNPKEPLISGWLFFRYLA
Tibetan_ground-tit_SERCA ALGFPEALIPVQLLWVNLVTDGLPATALGFNPPDLDIMNKPPRNPKEPLISGWLFFRYLA
Ground-finch_SERCA ALGFPEALIPVQLLWVNLVTDGLPATALGFNPPDLDIMNKPPRNPKEPLISGWLFFRYLA
Downy_woodpecker_SERCA ALGFPEALIPVQLLWVNLVTDGLPATALGFNPPDLDIMNKPPRNPKEPLISGWLFFRYLA
Struthio_camelus_SERCA ALGFPEALIPVQLLWVNLVTDGLPATALGFNPPDLDIMNKPPRNPKEPLISGWLFFRYLA
Penguin_SERCA ALGFPEALIPVQLLWVNLVTDGLPATALGFNPPDLDIMNKPPRNPKEPLISGWLFFRYLA
Adelie_penguin_SERCA ALGFPEALIPVQLLWVNLVTDGLPATALGFNPPDLDIMNKPPRNPKEPLISGWLFFRYLA
Ruber_ruber_SERCA ALGFPEALIPVQLLWVNLVTDGLPATALGFNPPDLDIMNKPPRNPKEPLISGWLFFRYLA
Swift_SERCA ALGFPEALIPVQLLWVNLVTDGLPATALGFNPPDLDIMNKPPRNPKEPLISGWLFFRYLA
Falcon_SERCA ALGFPEALIPVQLLWVNLVTDGLPATALGFNPPDLDIMNKPPRNPKEPLISGWLFFRYLA
Japanese-quail_SERCA ALGFPEALIPVQLLWVNLVTDGLPATALGFNPPDLDIMNKPPRNPKEPLISGWLFFRYLA
White-tailed_tropicbird_SERCA ALGFPEALIPVQLLWVNLVTDGLPATALGFNPPDLDIMNKPPRNPKEPLISGWLFFRYLA
Ruff_SERCA ALGFPEALIPVQLLWVNLVTDGLPATALGFNPPDLDIMNKPPRNPKEPLISGWLFFRYLA
Turkey_SERCA ALGFPEALIPVQLLWVNLVTDGLPATALGFNPPDLDIMNKPPRNPKEPLISGWLFFRYLA
Little-egret_SERCA ALGFPEALIPVQLLWVNLVTDGLPATALGFNPPDLDIMNKPPRNPKEPLISGWLFFRYLA
Cuckoo_SERCA ALGFPEALIPVQLLWVNLVTDGLPATALGFNPPDLDIMNKPPRNPKEPLISGWLFFRYLA
Crowned-crane_SERCA ALGFPEALIPVQLLWVNLVTDGLPATALGFNPPDLDIMNKPPRNPKEPLISGWLFFRYLA
Chicken_SERCA1 ALGLPEALIPVQLLWVNLVTDGLPATALGFNPPDLDIMDKPPRSPKEPLISGWLFFRYLA
Sifaka_SERCA1 ALGLPEALIPVQLLWVNLVTDGLPATALGFNPPDLDIMDRPPRSPKEPLISGWLFFRYMA
Pongo_SERCA1 ALGLPEALIPVQLLWVNLVTDGLPATALGFNPPDLDIMDRPPRSPKEPLISGWLFFRYMA
Human_SERCA1 ALGLPEALIPVQLLWVNLVTDGLPATALGFNPPDLDIMDRPPRSPKEPLISGWLFFRYMA
Chimpanzee_SERCA1 ALGLPEALIPVQLLWVNLVTDGLPATALGFNPPDLDIMDRPPRSPKEPLISGWLFFRYMA
Domestic_ferret_SERCA1 ALGLPEALIPVQLLWVNLVTDGLPATALGFNPPDLDIMDRPPRSPKEPLISGWLFFRYMA
Dog_SERCA1 ALGLPEALIPVQLLWVNLVTDGLPATALGFNPPDLDIMDRPPRSPKEPLISGWLFFRYMA
Panthera_SERCA1 ALGLPEALIPVQLLWVNLVTDGLPATALGFNPPDLDIMDRPPRSPKEPLISGWLFFRYMA
Cat_SERCA1 ALGLPEALIPVQLLWVNLVTDGLPATALGFNPPDLDIMDRPPRSPKEPLISGWLFFRYMA
Horseshoe-bat_SERCA1 ALGLPEALIPVQLLWVNLVTDGLPATALGFNPPDLDIMDRPPRSPKEPLISGWLFFRYMA
Vampire_bat_SERCA1 ALGLPEALIPVQLLWVNLVTDGLPATALGFNPPDLDIMDRPPRSPKEPLISGWLFFRYMA
Egyptian_rousette_SERCA1 ALGLPEALIPVQLLWVNLVTDGLPATALGFNPPDLDIMDRPPRSPKEPLISGWLFFRYMA
Philippine_tarsier_SERCA1 ALGLPEALIPVQLLWVNLVTDGLPATALGFNPPDLDIMDRPPRSPKEPLISGWLFFRYMA
Rabbit_SERCA1 ALGLPEALIPVQLLWVNLVTDGLPATALGFNPPDLDIMDRPPRSPKEPLISGWLFFRYMA
American_pika_SERCA1 ALGLPEALIPVQLLWVNLVTDGLPATALGFNPPDLDIMDRPPRSPKEPLISGWLFFRYMA
Thirteen-lined_ground_squirrel_SERCA1 ALGLPEALIPVQLLWVNLVTDGLPATALGFNPPDLDIMDRPPRSPKEPLISGWLFFRYMA
Alpine_marmot_SERCA1 ALGLPEALIPVQLLWVNLVTDGLPATALGFNPPDLDIMDRPPRSPKEPLISGWLFFRYMA
Chinchilla_SERCA1 ALGLPEALIPVQLLWVNLVTDGLPATALGFNPPDLDIMDRPPRSPKEPLISGWLFFRYMA
Cape_elephant-shrew_SERCA1 ALGLPEALIPVQLLWVNLVTDGLPATALGFNPPDLDIMDRPPRSPKEPLISGWLFFRYMA
Chinese_ ALGLPEALIPVQLLWVNLVTDGLPATALGFNPPDLDIMDRPPRSPKEPLISGWLFFRYMA
Kangaroo_rat_SERCA1 ALGLPEALIPVQLLWVNLVTDGLPATALGFNPPDLDIMDRPPRSPKEPLISGWLFFRYMA
Egyptian_jerboa_SERCA1 ALGLPEALIPVQLLWVNLVTDGLPATALGFNPPDLDIMDRPPRSPKEPLISGWLFFRYMA
Mongolian_gerbil_SERCA1 ALGLPEALIPVQLLWVNLVTDGLPATALGFNPPDLDIMDRPPRSPKEPLISGWLFFRYMA
Prairie_vole_SERCA1 ALGLPEALIPVQLLWVNLVTDGLPATALGFNPPDLDIMDRPPRSPKEPLISGWLFFRYMA
Chinese ALGLPEALIPVQLLWVNLVTDGLPATALGFNPPDLDIMDRPPRSPKEPLISGWLFFRYMA
Rattus_SERCA1 ALGLPEALIPVQLLWVNLVTDGLPATALGFNPPDLDIMDRPPRSPKEPLISGWLFFRYMA
Mus_SERCA1 ALGLPEALIPVQLLWVNLVTDGLPATALGFNPPDLDIMDRPPRSPKEPLISGWLFFRYMA

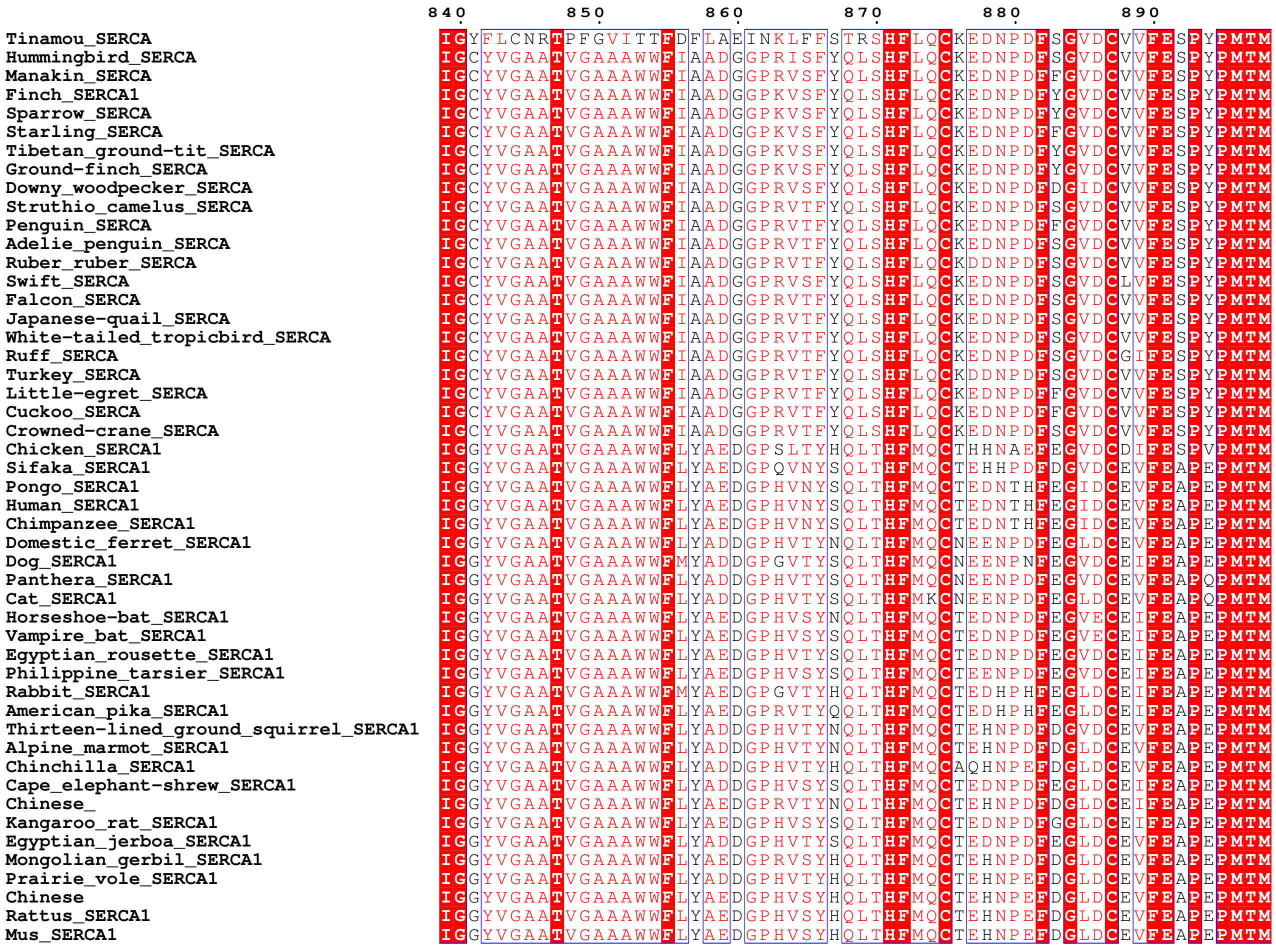
840 850 860 870 880 890
Tinamou_SERCA IGYFLCNRTPFGVITTFDFLAEINKLFFSTRSHFLQCKEDNPDFSGVDCVVFESPYPMTM
Hummingbird_SERCA IGCYVGAATVGAAAWWFIAADGGPRISFYQLSHFLQCKEDNPDFSGVDCVVFESPYPMTM
Manakin_SERCA IGCYVGAATVGAAAWWFIAADGGPRVSFYQLSHFLQCKEDNPDFFGVDCVVFESPYPMTM
Finch_SERCA1 IGCYVGAATVGAAAWWFIAADGGPKVSFYQLSHFLQCKEDNPDFYGVDCVVFESPYPMTM
Sparrow_SERCA IGCYVGAATVGAAAWWFIAADGGPKVSFYQLSHFLQCKEDNPDFYGVDCVVFESPYPMTM
Starling_SERCA IGCYVGAATVGAAAWWFIAADGGPKVSFYQLSHFLQCKEDNPDFFGVDCVVFESPYPMTM
Tibetan_ground-tit_SERCA IGCYVGAATVGAAAWWFIAADGGPKVSFYQLSHFLQCKEDNPDFYGVDCVVFESPYPMTM
Ground-finch_SERCA IGCYVGAATVGAAAWWFIAADGGPKVSFYQLSHFLQCKEDNPDFYGVDCVVFESPYPMTM
Downy_woodpecker_SERCA IGCYVGAATVGAAAWWFIAADGGPRVSFYQLSHFLQCKEDNPDFDGIDCVVFESPYPMTM
Struthio_camelus_SERCA IGCYVGAATVGAAAWWFIAADGGPRVTFYQLSHFLQCKEDNPDFSGVDCVVFESPYPMTM
Penguin_SERCA IGCYVGAATVGAAAWWFIAADGGPRVTFYQLSHFLQCKEDNPDFFGVDCVVFESPYPMTM
Adelie_penguin_SERCA IGCYVGAATVGAAAWWFIAADGGPRVTFYQLSHFLQCKEDNPDFSGVDCVVFESPYPMTM
Ruber_ruber_SERCA IGCYVGAATVGAAAWWFIAADGGPRVTFYQLSHFLQCKDDNPDFSGVDCVVFESPYPMTM
Swift_SERCA IGCYVGAATVGAAAWWFIAADGGPRVSFYQLSHFLQCKEDNPDFSGVDCLVFESPYPMTM
Falcon_SERCA IGCYVGAATVGAAAWWFIAADGGPRVTFYQLSHFLQCKEDNPDFSGVDCVVFESPYPMTM
Japanese-quail_SERCA IGCYVGAATVGAAAWWFIAADGGPRVTFYQLSHFLQCKEDNPDFSGVDCVVFESPYPMTM
White-tailed_tropicbird_SERCA IGCYVGAATVGAAAWWFIAADGGPRVTFYQLSHFLQCKEDNPDFSGVDCVVFESPYPMTM
Ruff_SERCA IGCYVGAATVGAAAWWFIAADGGPRVTFYQLSHFLQCKEDNPDFSGVDCGIFESPYPMTM
Turkey_SERCA IGCYVGAATVGAAAWWFIAADGGPRVTFYQLSHFLQCKDDNPDFSGVDCVVFESPYPMTM
Little-egret_SERCA IGCYVGAATVGAAAWWFIAADGGPRVTFYQLSHFLQCKEDNPDFFGVDCVVFESPYPMTM
Cuckoo_SERCA IGCYVGAATVGAAAWWFIAADGGPRVTFYQLSHFLQCKEDNPDFFGVDCVVFESPYPMTM
Crowned-crane_SERCA IGCYVGAATVGAAAWWFIAADGGPRVTFYQLSHFLQCKEDNPDFSGVDCVVFESPYPMTM
Chicken_SERCA1 IGGYVGAATVGAAAWWFLYAEDGPSLTYHQLTHFMQCTHHNAEFEGVDCDIFESPVPMTM
Sifaka_SERCA1 IGGYVGAATVGAAAWWFLYAEDGPQVNYSQLTHFMQCTEHHPDFDGVDCEVFEAPEPMTM
Pongo_SERCA1 IGGYVGAATVGAAAWWFLYAEDGPHVNYSQLTHFMQCTEDNTHFEGIDCEVFEAPEPMTM
Human_SERCA1 IGGYVGAATVGAAAWWFLYAEDGPHVNYSQLTHFMQCTEDNTHFEGIDCEVFEAPEPMTM
Chimpanzee_SERCA1 IGGYVGAATVGAAAWWFLYAEDGPHVNYSQLTHFMQCTEDNTHFEGIDCEVFEAPEPMTM
Domestic_ferret_SERCA1 IGGYVGAATVGAAAWWFLYADDGPHVTYNQLTHFMQCNEENPDFEGLDCEVFEAPEPMTM
Dog_SERCA1 IGGYVGAATVGAAAWWFMYADDGPGVTYSQLTHFMQCNEENPNFEGVDCEIFEAPEPMTM
Panthera_SERCA1 IGGYVGAATVGAAAWWFLYADDGPHVTYSQLTHFMQCNEENPDFEGVDCEVFEAPQPMTM
Cat_SERCA1 IGGYVGAATVGAAAWWFLYADDGPHVTYSQLTHFMKCNEENPDFEGLDCEVFEAPQPMTM
Horseshoe-bat_SERCA1 IGGYVGAATVGAAAWWFLYAEDGPHVSYNQLTHFMQCTEDNPDFEGVECEIFEAPEPMTM
Vampire_bat_SERCA1 IGGYVGAATVGAAAWWFLYAEDGPHVSYSQLTHFMQCTEDNPDFEGVECEIFEAPEPMTM
Egyptian_rousette_SERCA1 IGGYVGAATVGAAAWWFLYAEDGPHVTYSQLTHFMQCTEDNPDFEGVDCEIFEAPEPMTM
Philippine_tarsier_SERCA1 IGGYVGAATVGAAAWWFLYAEDGPHVSYSQLTHFMQCTEENPDFEGVDCEIFEAPEPMTM
Rabbit_SERCA1 IGGYVGAATVGAAAWWFMYAEDGPGVTYHQLTHFMQCTEDHPHFEGLDCEIFEAPEPMTM
American_pika_SERCA1 IGGYVGAATVGAAAWWFLYAEDGPRVTYQQLTHFMQCTEDHPHFEGLDCEIFEAPEPMTM
Thirteen-lined_ground_squirrel_SERCA1 IGGYVGAATVGAAAWWFLYADDGPHVTYNQLTHFMQCTEHNPDFDGVDCEVFEAPEPMTM
Alpine_marmot_SERCA1 IGGYVGAATVGAAAWWFLYADDGPHVTYNQLTHFMQCTEHNPDFDGLDCEVFEAPEPMTM
Chinchilla_SERCA1 IGGYVGAATVGAAAWWFLYADDGPHVTYHQLTHFMQCAQHNPEFDGLDCEVFEAPEPMTM
Cape_elephant-shrew_SERCA1 IGGYVGAATVGAAAWWFLYADDGPHVSYSQLTHFMQCTEDNPDFEGLDCEIFEAPEPMTM
Chinese_ IGGYVGAATVGAAAWWFLYAEDGPRVTYNQLTHFMQCTEHNPDFDGLDCEIFEAPEPMTM
Kangaroo_rat_SERCA1 IGGYVGAATVGAAAWWFLYAEDGPHVSYSQLTHFMQCTEDNPDFGGLDCEIFEAPEPMTM
Egyptian_jerboa_SERCA1 IGGYVGAATVGAAAWWFLYADDGPHVTYSQLTHFMQCTEDNPDFEGLDCEVFEAPEPMTM
Mongolian_gerbil_SERCA1 IGGYVGAATVGAAAWWFLYAEDGPRVSYHQLTHFMQCTEHNPDFDGLDCEVFEAPEPMTM
Prairie_vole_SERCA1 IGGYVGAATVGAAAWWFLYADDGPHVTYHQLTHFMQCTEHNPEFDGLDCEVFEAPEPMTM
Chinese IGGYVGAATVGAAAWWFLYAEDGPHVSYHQLTHFMQCTEHNPEFDGLDCEVFEAPEPMTM
Rattus_SERCA1 IGGYVGAATVGAAAWWFLYAEDGPHVSYHQLTHFMQCTEHNPEFDGLDCEVFEAPEPMTM
Mus_SERCA1 IGGYVGAATVGAAAWWFLYAEDGPHVSYHQLTHFMQCTEHNPEFDGLDCEVFEAPEPMTM

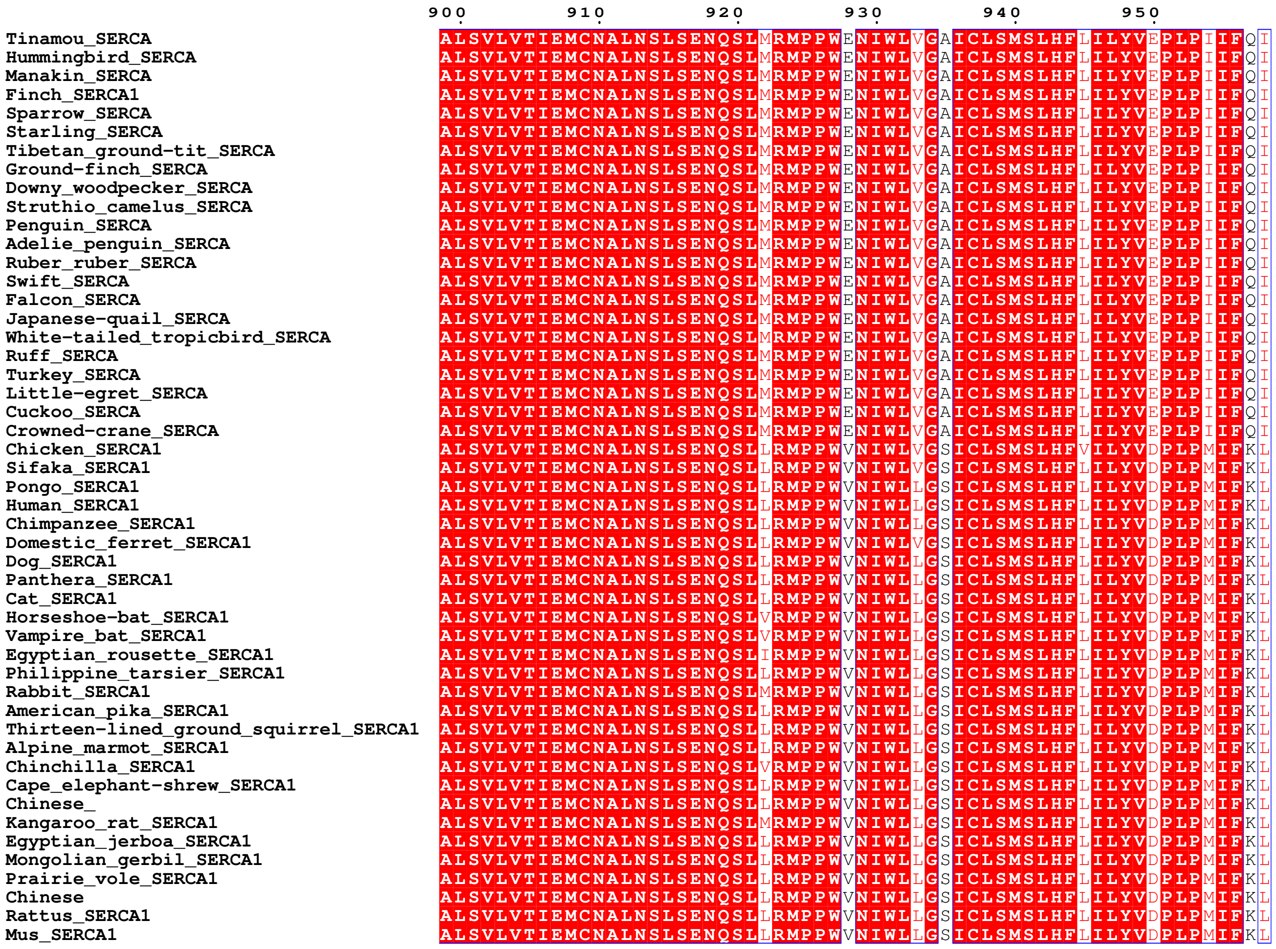
900 910 920 930 940 950
Tinamou_SERCA ALSVLVTIEMCNALNSLSENQSLMRMPPWENIWLVGAICLSMSLHFLILYVEPLPIIFQI
Hummingbird_SERCA ALSVLVTIEMCNALNSLSENQSLMRMPPWENIWLVGAICLSMSLHFLILYVEPLPIIFQI
Manakin_SERCA ALSVLVTIEMCNALNSLSENQSLMRMPPWENIWLVGAICLSMSLHFLILYVEPLPIIFQI
Finch_SERCA1 ALSVLVTIEMCNALNSLSENQSLMRMPPWENIWLVGAICLSMSLHFLILYVEPLPIIFQI
Sparrow_SERCA ALSVLVTIEMCNALNSLSENQSLMRMPPWENIWLVGAICLSMSLHFLILYVEPLPIIFQI
Starling_SERCA ALSVLVTIEMCNALNSLSENQSLMRMPPWENIWLVGAICLSMSLHFLILYVEPLPIIFQI
Tibetan_ground-tit_SERCA ALSVLVTIEMCNALNSLSENQSLMRMPPWENIWLVGAICLSMSLHFLILYVEPLPIIFQI
Ground-finch_SERCA ALSVLVTIEMCNALNSLSENQSLMRMPPWENIWLVGAICLSMSLHFLILYVEPLPIIFQI
Downy_woodpecker_SERCA ALSVLVTIEMCNALNSLSENQSLMRMPPWENIWLVGAICLSMSLHFLILYVEPLPIIFQI
Struthio_camelus_SERCA ALSVLVTIEMCNALNSLSENQSLMRMPPWENIWLVGAICLSMSLHFLILYVEPLPIIFQI
Penguin_SERCA ALSVLVTIEMCNALNSLSENQSLMRMPPWENIWLVGAICLSMSLHFLILYVEPLPIIFQI
Adelie_penguin_SERCA ALSVLVTIEMCNALNSLSENQSLMRMPPWENIWLVGAICLSMSLHFLILYVEPLPIIFQI
Ruber_ruber_SERCA ALSVLVTIEMCNALNSLSENQSLMRMPPWENIWLVGAICLSMSLHFLILYVEPLPIIFQI
Swift_SERCA ALSVLVTIEMCNALNSLSENQSLMRMPPWENIWLVGAICLSMSLHFLILYVEPLPIIFQI
Falcon_SERCA ALSVLVTIEMCNALNSLSENQSLMRMPPWENIWLVGAICLSMSLHFLILYVEPLPIIFQI
Japanese-quail_SERCA ALSVLVTIEMCNALNSLSENQSLMRMPPWENIWLVGAICLSMSLHFLILYVEPLPIIFQI
White-tailed_tropicbird_SERCA ALSVLVTIEMCNALNSLSENQSLMRMPPWENIWLVGAICLSMSLHFLILYVEPLPIIFQI
Ruff_SERCA ALSVLVTIEMCNALNSLSENQSLMRMPPWENIWLVGAICLSMSLHFLILYVEPLPIIFQI
Turkey_SERCA ALSVLVTIEMCNALNSLSENQSLMRMPPWENIWLVGAICLSMSLHFLILYVEPLPIIFQI
Little-egret_SERCA ALSVLVTIEMCNALNSLSENQSLMRMPPWENIWLVGAICLSMSLHFLILYVEPLPIIFQI
Cuckoo_SERCA ALSVLVTIEMCNALNSLSENQSLMRMPPWENIWLVGAICLSMSLHFLILYVEPLPIIFQI
Crowned-crane_SERCA ALSVLVTIEMCNALNSLSENQSLMRMPPWENIWLVGAICLSMSLHFLILYVEPLPIIFQI
Chicken_SERCA1 ALSVLVTIEMCNALNSLSENQSLLRMPPWVNIWLVGSICLSMSLHFVILYVDPLPMIFKL
Sifaka_SERCA1 ALSVLVTIEMCNALNSLSENQSLLRMPPWVNIWLVGSICLSMSLHFLILYVDPLPMIFKL
Pongo_SERCA1 ALSVLVTIEMCNALNSLSENQSLLRMPPWVNIWLLGSICLSMSLHFLILYVDPLPMIFKL
Human_SERCA1 ALSVLVTIEMCNALNSLSENQSLLRMPPWVNIWLLGSICLSMSLHFLILYVDPLPMIFKL
Chimpanzee_SERCA1 ALSVLVTIEMCNALNSLSENQSLLRMPPWVNIWLLGSICLSMSLHFLILYVDPLPMIFKL
Domestic_ferret_SERCA1 ALSVLVTIEMCNALNSLSENQSLLRMPPWVNIWLVGSICLSMSLHFLILYVDPLPMIFKL
Dog_SERCA1 ALSVLVTIEMCNALNSLSENQSLLRMPPWVNIWLLGSICLSMSLHFLILYVDPLPMIFKL
Panthera_SERCA1 ALSVLVTIEMCNALNSLSENQSLLRMPPWVNIWLLGSICLSMSLHFLILYVDPLPMIFKL
Cat_SERCA1 ALSVLVTIEMCNALNSLSENQSLLRMPPWVNIWLLGSICLSMSLHFLILYVDPLPMIFKL
Horseshoe-bat_SERCA1 ALSVLVTIEMCNALNSLSENQSLVRMPPWVNIWLLGSICLSMSLHFLILYVDPLPMIFKL
Vampire_bat_SERCA1 ALSVLVTIEMCNALNSLSENQSLVRMPPWVNIWLLGSICLSMSLHFLILYVDPLPMIFKL
Egyptian_rousette_SERCA1 ALSVLVTIEMCNALNSLSENQSLIRMPPWVNIWLLGSICLSMSLHFLILYVDPLPMIFKL
Philippine_tarsier_SERCA1 ALSVLVTIEMCNALNSLSENQSLLRMPPWVNIWLLGSICLSMSLHFLILYVDPLPMIFKL
Rabbit_SERCA1 ALSVLVTIEMCNALNSLSENQSLMRMPPWVNIWLLGSICLSMSLHFLILYVDPLPMIFKL
American_pika_SERCA1 ALSVLVTIEMCNALNSLSENQSLLRMPPWVNIWLLGSICLSMSLHFLILYVDPLPMIFKL
Thirteen-lined_ground_squirrel_SERCA1 ALSVLVTIEMCNALNSLSENQSLLRMPPWVNIWLLGSICLSMSLHFLILYVDPLPMIFKL
Alpine_marmot_SERCA1 ALSVLVTIEMCNALNSLSENQSLLRMPPWVNIWLLGSICLSMSLHFLILYVDPLPMIFKL
Chinchilla_SERCA1 ALSVLVTIEMCNALNSLSENQSLVRMPPWVNIWLLGSICLSMSLHFLILYVDPLPMIFKL
Cape_elephant-shrew_SERCA1 ALSVLVTIEMCNALNSLSENQSLLRMPPWVNIWLLGSICLSMSLHFLILYVDPLPMIFKL
Chinese_ ALSVLVTIEMCNALNSLSENQSLLRMPPWVNIWLLGSICLSMSLHFLILYVDPLPMIFKL
Kangaroo_rat_SERCA1 ALSVLVTIEMCNALNSLSENQSLMRMPPWVNIWLLGSICLSMSLHFLILYVDPLPMIFKL
Egyptian_jerboa_SERCA1 ALSVLVTIEMCNALNSLSENQSLLRMPPWVNIWLLGSICLSMSLHFLILYVDPLPMIFKL
Mongolian_gerbil_SERCA1 ALSVLVTIEMCNALNSLSENQSLLRMPPWVNIWLLGSICLSMSLHFLILYVDPLPMIFKL
Prairie_vole_SERCA1 ALSVLVTIEMCNALNSLSENQSLLRMPPWVNIWLLGSICLSMSLHFLILYVDPLPMIFKL
Chinese ALSVLVTIEMCNALNSLSENQSLLRMPPWVNIWLLGSICLSMSLHFLILYVDPLPMIFKL
Rattus_SERCA1 ALSVLVTIEMCNALNSLSENQSLLRMPPWVNIWLLGSICLSMSLHFLILYVDPLPMIFKL
Mus_SERCA1 ALSVLVTIEMCNALNSLSENQSLLRMPPWVNIWLLGSICLSMSLHFLILYVDPLPMIFKL

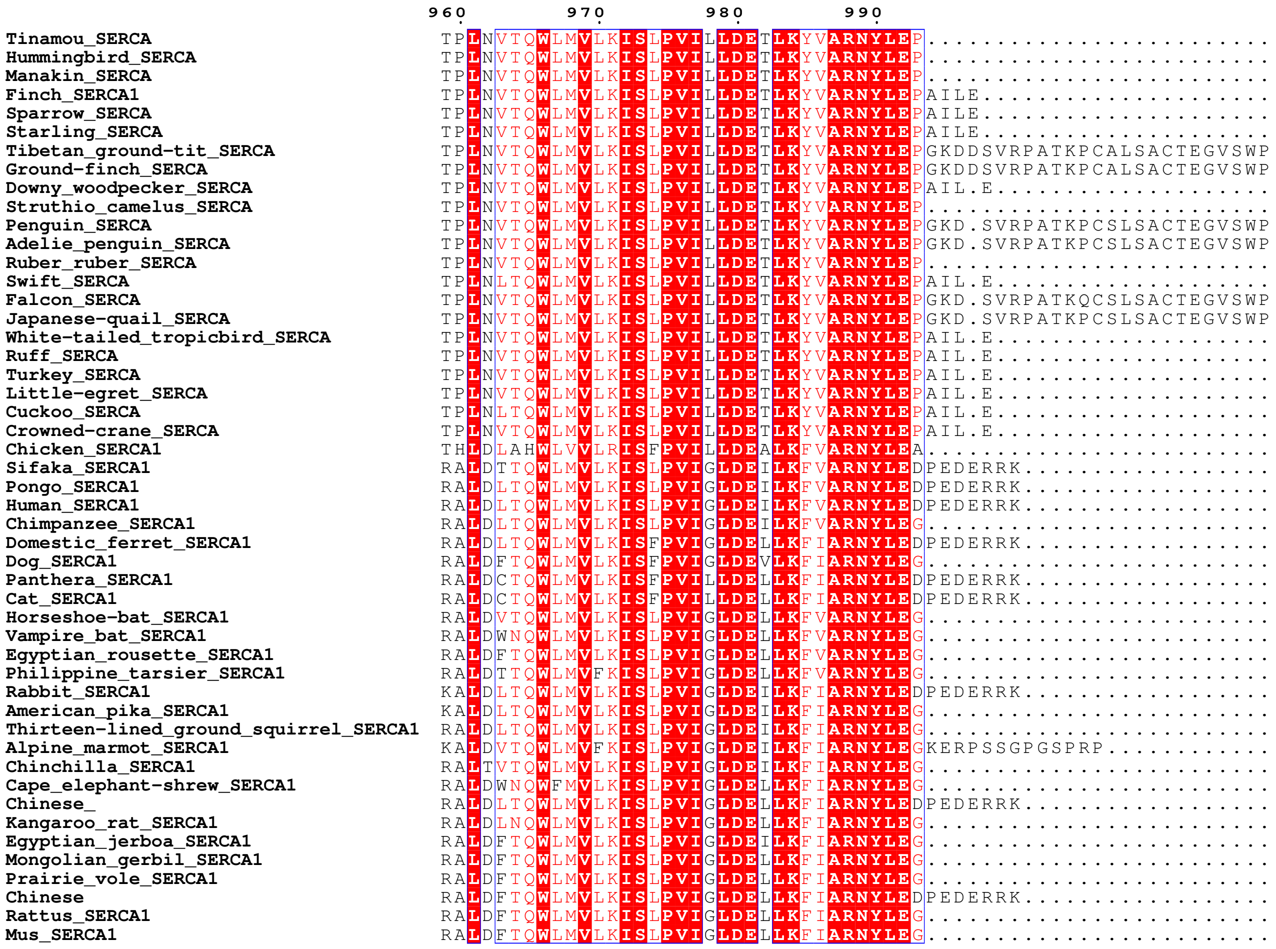
960 970 980 990
Tinamou_SERCA TPLNVTQWLMVLKISLPVILLDETLKYVARNYLEP.........................
Hummingbird_SERCA TPLNVTQWLMVLKISLPVILLDETLKYVARNYLEP.........................
Manakin_SERCA TPLNVTQWLMVLKISLPVILLDETLKYVARNYLEP.........................
Finch_SERCA1 TPLNVTQWLMVLKISLPVILLDETLKYVARNYLEPAILE.....................
Sparrow_SERCA TPLNVTQWLMVLKISLPVILLDETLKYVARNYLEPAILE.....................
Starling_SERCA TPLNVTQWLMVLKISLPVILLDETLKYVARNYLEPAILE.....................
Tibetan_ground-tit_SERCA TPLNVTQWLMVLKISLPVILLDETLKYVARNYLEPGKDDSVRPATKPCALSACTEGVSWP
Ground-finch_SERCA TPLNVTQWLMVLKISLPVILLDETLKYVARNYLEPGKDDSVRPATKPCALSACTEGVSWP
Downy_woodpecker_SERCA TPLNVTQWLMVLKISLPVILLDETLKYVARNYLEPAIL.E....................
Struthio_camelus_SERCA TPLNVTQWLMVLKISLPVILLDETLKYVARNYLEP.........................
Penguin_SERCA TPLNVTQWLMVLKISLPVILLDETLKYVARNYLEPGKD.SVRPATKPCSLSACTEGVSWP
Adelie_penguin_SERCA TPLNVTQWLMVLKISLPVILLDETLKYVARNYLEPGKD.SVRPATKPCSLSACTEGVSWP
Ruber_ruber_SERCA TPLNVTQWLMVLKISLPVILLDETLKYVARNYLEP.........................
Swift_SERCA TPLNLTQWLMVLKISLPVILLDETLKYVARNYLEPAIL.E....................
Falcon_SERCA TPLNVTQWLMVLKISLPVILLDETLKYVARNYLEPGKD.SVRPATKQCSLSACTEGVSWP
Japanese-quail_SERCA TPLNVTQWLMVLKISLPVILLDETLKYVARNYLEPGKD.SVRPATKPCSLSACTEGVSWP
White-tailed_tropicbird_SERCA TPLNVTQWLMVLKISLPVILLDETLKYVARNYLEPAIL.E....................
Ruff_SERCA TPLNVTQWLMVLKISLPVILLDETLKYVARNYLEPAIL.E....................
Turkey_SERCA TPLNVTQWLMVLKISLPVILLDETLKYVARNYLEPAIL.E....................
Little-egret_SERCA TPLNVTQWLMVLKISLPVILLDETLKYVARNYLEPAIL.E....................
Cuckoo_SERCA TPLNLTQWLMVLKISLPVILLDETLKYVARNYLEPAIL.E....................
Crowned-crane_SERCA TPLNVTQWLMVLKISLPVILLDETLKYVARNYLEPAIL.E....................
Chicken_SERCA1 THLDLAHWLVVLRISFPVILLDEALKFVARNYLEA.........................
Sifaka_SERCA1 RALDTTQWLMVLKISLPVIGLDEILKFVARNYLEDPEDERRK..................
Pongo_SERCA1 RALDLTQWLMVLKISLPVIGLDEILKFVARNYLEDPEDERRK..................
Human_SERCA1 RALDLTQWLMVLKISLPVIGLDEILKFVARNYLEDPEDERRK..................
Chimpanzee_SERCA1 RALDLTQWLMVLKISLPVIGLDEILKFVARNYLEG.........................
Domestic_ferret_SERCA1 RALDLTQWLMVLKISFPVIGLDELLKFIARNYLEDPEDERRK..................
Dog_SERCA1 RALDFTQWLMVLKISFPVIGLDEVLKFIARNYLEG.........................
Panthera_SERCA1 RALDCTQWLMVLKISFPVILLDELLKFIARNYLEDPEDERRK..................
Cat_SERCA1 RALDCTQWLMVLKISFPVILLDELLKFIARNYLEDPEDERRK..................
Horseshoe-bat_SERCA1 RALDVTQWLMVLKISLPVIGLDELLKFVARNYLEG.........................
Vampire_bat_SERCA1 RALDWNQWLMVLKISLPVIGLDELLKFVARNYLEG.........................
Egyptian_rousette_SERCA1 RALDFTQWLMVLKISLPVIGLDELLKFVARNYLEG.........................
Philippine_tarsier_SERCA1 RALDTTQWLMVFKISLPVIGLDELLKFVARNYLEG.........................
Rabbit_SERCA1 KALDLTQWLMVLKISLPVIGLDEILKFIARNYLEDPEDERRK..................
American_pika_SERCA1 KALDLTQWLMVLKISLPVIGLDEILKFIARNYLEG.........................
Thirteen-lined_ground_squirrel_SERCA1 RALDLTQWLMVLKISLPVIGLDEILKFIARNYLEG.........................
Alpine_marmot_SERCA1 KALDVTQWLMVFKISLPVIGLDEILKFIARNYLEGKERPSSGPGSPRP............
Chinchilla_SERCA1 RALTVTQWLMVLKISLPVIGLDEILKFIARNYLEG.........................
Cape_elephant-shrew_SERCA1 RALDWNQWFMVLKISLPVIGLDELLKFIARNYLEG.........................
Chinese_ RALDLTQWLMVLKISLPVIGLDELLKFIARNYLEDPEDERRK..................
Kangaroo_rat_SERCA1 RALDLNQWLMVLKISLPVIGLDELLKFIARNYLEG.........................
Egyptian_jerboa_SERCA1 RALDFTQWLMVLKISLPVIGLDEILKFIARNYLEG.........................
Mongolian_gerbil_SERCA1 RALDFTQWLMVLKISLPVIGLDELLKFIARNYLEG.........................
Prairie_vole_SERCA1 RALDFTQWLMVLKISLPVIGLDELLKFIARNYLEG.........................
Chinese RALDFTQWLMVLKISLPVIGLDELLKFIARNYLEDPEDERRK..................
Rattus_SERCA1 RALDFTQWLMVLKISLPVIGLDELLKFIARNYLEG.........................
Mus_SERCA1 RALDFTQWLMVLKISLPVIGLDELLKFIARNYLEG.........................

**Tinamou_SERCA**	....
**Hummingbird_SERCA**	....
**Manakin_SERCA**	....
**Finch_SERCA1**	....
**Sparrow_SERCA**	....
**Starling_SERCA**	....
**Tibetan_ground-tit_SERCA**	FVFITMPLVIWLYSTDTNFSDMFWS
**Ground-finch_SERCA**	FVFITLPLVIWLYSTDTNFSDMFWS
**Downy_woodpecker_SERCA**	....
**Struthio_camelus_SERCA**	....
**Penguin_SERCA**	FVFITVPLVIWLYSTDTNFSDMFWS
**Adelie_penguin_SERCA**	FVFITMPLVIWLYSTDTNFSDMFWS
**Ruber_ruber_SERCA**	....
**Swift_SERCA**	....
**Falcon_SERCA**	FVFITMPLVIWLYSTDTNFSDMFWS
**Japanese-quail_SERCA**	FVFITLPLVIWLYSTDTNFSDMFLS
**White-tailed_tropicbird_SERCA**	....
**Ruff_SERCA**	....
**Turkey_SERCA**	....
**Little-egret_SERCA**	....
**Cuckoo_SERCA**	....
**Crowned-crane_SERCA**	....
**Chicken_SERCA1**	....
**Sifaka_SERCA1**	....
**Pongo_SERCA1**	....
**Human_SERCA1**	....
**Chimpanzee_SERCA1**	....
**Domestic_ferret_SERCA1**	....
**Dog_SERCA1**	....
**Panthera_SERCA1**	....
**Cat_SERCA1**	....
**Horseshoe-bat_SERCA1**	....
**Vampire_bat_SERCA1**	....
**Egyptian_rousette_SERCA1**	....
**Philippine_tarsier_SERCA1**	....
**Rabbit_SERCA1**	....
**American_pika_SERCA1**	....
**Thirteen-lined_ground_squirrel_SERCA1**	....
**Alpine_marmot_SERCA1**	....
**Chinchilla_SERCA1**	....
**Cape_elephant-shrew_SERCA1**	....
**Chinese_**	....
**Kangaroo_rat_SERCA1**	....
**Egyptian_jerboa_SERCA1**	....
**Mongolian_gerbil_SERCA1**	....
**Prairie_vole_SERCA1**	....
**Chinese**	....
**Rattus_SERCA1**	....
**Mus_SERCA1**	....
